# GWAS identifies candidate genes controlling adventitious rooting in *Populus trichocarpa*

**DOI:** 10.1093/hr/uhad125

**Published:** 2023-06-14

**Authors:** Michael F Nagle, Jialin Yuan, Damanpreet Kaur, Cathleen Ma, Ekaterina Peremyslova, Yuan Jiang, Bahiya Zahl, Alexa Niño de Rivera, Wellington Muchero, Li Fuxin, Steven H Strauss

**Affiliations:** Department of Forest Ecosystems and Society, Oregon State University, 3180 SW Jefferson Way, Corvallis, OR, 97331, United States; Department of Electrical Engineering and Computer Science, Oregon State University, 110 SW Park Terrace, Corvallis, OR, 97331, United States; Department of Electrical Engineering and Computer Science, Oregon State University, 110 SW Park Terrace, Corvallis, OR, 97331, United States; Department of Forest Ecosystems and Society, Oregon State University, 3180 SW Jefferson Way, Corvallis, OR, 97331, United States; Department of Forest Ecosystems and Society, Oregon State University, 3180 SW Jefferson Way, Corvallis, OR, 97331, United States; Statistics Department, Oregon State University, 103 SW Memorial Place, Corvallis, OR, 97331, United States; Department of Forest Ecosystems and Society, Oregon State University, 3180 SW Jefferson Way, Corvallis, OR, 97331, United States; Department of Forest Ecosystems and Society, Oregon State University, 3180 SW Jefferson Way, Corvallis, OR, 97331, United States; Biosciences Division, Oak Ridge National Laboratory, 1 Bethel Valley Rd, Oak Ridge, TN, 37830, United States; Center for Bioenergy Innovation, Oak Ridge National Laboratory, 1 Bethel Valley Rd, Oak Ridge, TN, 37830, United States; Bredesen Center for Interdisciplinary Research, University of Tennessee, 821 Volunteer Blvd., Knoxville, TN, 37996, United States; Department of Electrical Engineering and Computer Science, Oregon State University, 110 SW Park Terrace, Corvallis, OR, 97331, United States; Department of Forest Ecosystems and Society, Oregon State University, 3180 SW Jefferson Way, Corvallis, OR, 97331, United States

## Abstract

Adventitious rooting (AR) is critical to the propagation, breeding, and genetic engineering of trees. The capacity for plants to undergo this process is highly heritable and of a polygenic nature; however, the basis of its genetic variation is largely uncharacterized. To identify genetic regulators of AR, we performed a genome-wide association study (GWAS) using 1148 genotypes of *Populus trichocarpa*. GWASs are often limited by the abilities of researchers to collect precise phenotype data on a high-throughput scale; to help overcome this limitation, we developed a computer vision system to measure an array of traits related to adventitious root development in poplar, including temporal measures of lateral and basal root length and area. GWAS was performed using multiple methods and significance thresholds to handle non-normal phenotype statistics and to gain statistical power. These analyses yielded a total of 277 unique associations, suggesting that genes that control rooting include regulators of hormone signaling, cell division and structure, reactive oxygen species signaling, and other processes with known roles in root development. Numerous genes with uncharacterized functions and/or cryptic roles were also identified. These candidates provide targets for functional analysis, including physiological and epistatic analyses, to better characterize the complex polygenic regulation of AR.

## Introduction

Species within the genus *Populus* (poplar) are among the most rapidly growing trees of the northern hemisphere and have keystone roles in many natural ecosystems. They are also of major economic importance for agroforestry and as sources of wood, fiber, and biofuel [1]. The growth and asexual propagation of poplar relies on the rapid establishment, proliferation, and maintenance of a robust root system for nutrient and water absorption. Elite hybrid clones of poplar are propagated in stool beds through a process that relies on the ability of cuttings to undergo adventitious rooting (AR) [2]. A deeper understanding of the genes that control rooting may provide new insights into means for improved propagation of recalcitrant genotypes and species, provide options for improvement of regeneration during genetic engineering/editing, and suggest new strategies for mitigating stress in managed and wild populations.

AR in poplar is a highly complex trait that is regulated by many factors, including plant age, genotype, and physiology; the many forms of plant stress; and environmental cues such as temperature, photoperiod, and nutrients. The effects of these variables are mediated via phytohormone signaling cascades that lead to differentiation and development of root tissue. Overexpression or RNAi-mediated suppression of over 20 genes, spanning phytohormone pathways, including auxin, cytokinin, gibberellin, and jasmonate (JA) signaling, has been reported to lead to increased or decreased AR and/or root growth in poplar (recently reviewed [3]). Bioinformatic approaches including transcriptomic analysis and quantitative trait locus (QTL) mapping have contributed to an improved understanding of how these pathways and others underlie variation in abilities of diverse poplar genotypes to undergo AR, underscoring the importance of phytohormone synthesis and signaling, among other diverse processes [4, 5]. Advancements in the understanding of variation in AR response across genotypes may support the development of hormonal or transgenic treatments to improve AR in recalcitrant genotypes.

Measurement of root traits to be studied by association mapping approaches such as QTL mapping or genome-wide association studies (GWASs) can be highly complex, as roots can be phenotyped with respect to not only spatial features such as their lengths and area but also their branching patterns and/or origins. These measurements can prove laborious and time-consuming if done manually. A wide array of computer vision tools have been developed to collect measurements from root images, with varying degrees of human intervention or automation across a diverse range of plants [6–8] (recently reviewed [9, 10]), including poplar [5]. The production of general and user-friendly root phenotyping platforms can help to extend automated methods to more species and laboratories, enabling larger population sizes and increased statistical power by reducing the labor needed for phenotyping.

**Figure 1:**
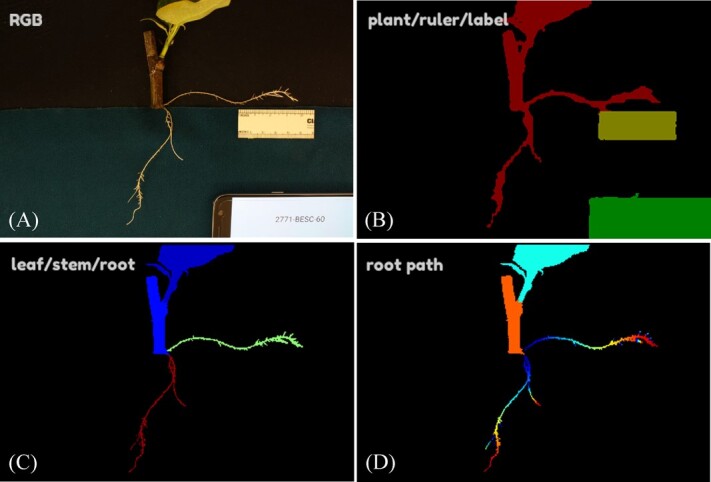
Workflow for phenotyping root traits: (A) RGB images were collected for plants with rulers and labels. (B) The first round of deep segmentation was performed to separate the plant (red), ruler (yellow), and label (green). (C) A subsequent round of deep segmentation and additional processing, including connected components and thresholding, distinguished types of plant tissue, including leaf (dark blue), stem (lighter blue), lateral root (green), and basal root (red). (D) Next, the length of each root was measured as the shortest path from root tip to their point of origin, whether originating from another root or from stem. Distance of a given point from the origin of a given root is indicated by a spectrum from blue (near origin) to red (far from origin). Final outputs are summary statistics for total area and LRL computed over basal and lateral types of AR. Examples of images producing errors are given in Fig. S1.

Here, we report insights into the genetic control of rooting obtained through GWAS. Using a large resequenced and highly polymorphic population of wild *Populus trichocarpa*, a novel computer vision phenomic system (Fig. 1), and multiple GWAS approaches, we were able to detect 277 statistically significant associations. These implicate genes involved in hormone signaling, cell division and expansion, reactive oxygen species (ROS) regulation, and post-translational modification (PTM) of proteins—in addition to many genes of unknown function. Our results indicate that variation in AR is highly polygenic and that large numbers of genes contribute minor effects.

## Results

### Principal components describe complex patterns of root development

Significant patterns of root development over time and across root types (basal and lateral) were summarized using principal component analysis (PCA). For each of the three groups of traits used for PCA, the top two principal components (PCs) appear to explain significant portions of variance as indicated by scree plots. PCA was performed over three groups as described (Materials and methods). The first two groups, for root area or root length traits, each across basal and lateral roots and time points, both produced first and second PCs (PC1 and PC2) representing contrasts between basal and lateral root development. The combined variance explained by PC1 and PC2 is ~92% for the group with root area traits and ~ 90% for the group with root length traits (Figs S2–S3). For the third group, including area and length traits together, PC1 shows a trend of overall root development, PC2 represents a basal/lateral contrast, and the combined variance explained by PC1 and PC2 is ~75% (Fig. S4).

### Use of multiple GWAS methods yielded numerous associations

**Figure 2:**
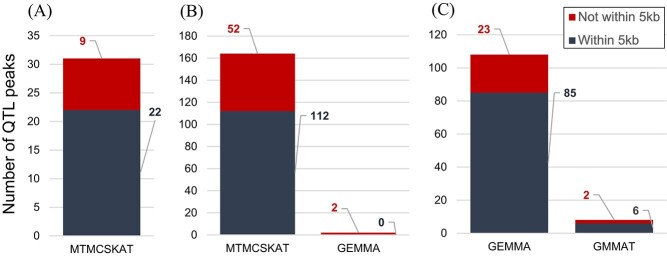
Barplots summarizing the numbers of associations from each GWAS method (*x*-axis labels), with each type of significance threshold, as well as within 5 kb of the nearest gene. QTL peaks were taken as the lowest *P* value at any given position near a significant SNP, where multiple points within the same peak may pass a given significance threshold. (A) QTL peaks passing the conservative Bonferroni threshold (*α* = 0.05; *N* tests equal to *N* SNPs for GEMMA/GMMAT or *N* SNP windows for SKAT). (B) QTL peaks passing Benjamini–Hochberg FDR threshold (*α* = 0.10) and/or the conservative Bonferroni threshold. (C) QTL peaks passing ART–Bonferroni threshold (*α* = 0.05, *N* of 1 kb windows in genome); ART was only applied to the single-SNP GWAS methods, GEMMA and GMMAT. This figure provides the counts of unique associations, such that candidate genes appearing across multiple traits are only counted once for a given GWAS method and threshold.

**Figure 3:**
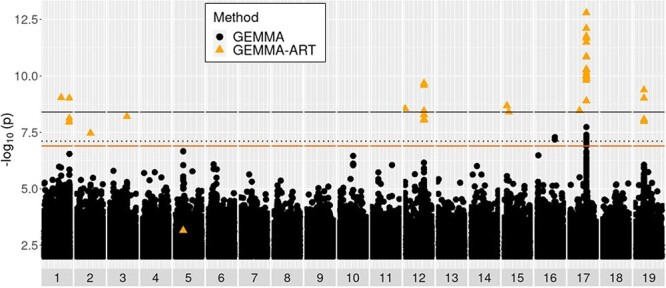
Manhattan plot of GEMMA results for the trait of longest lateral root length at Week 3. Black and orange solid lines represent Bonferroni significance thresholds for GEMMA results with independent SNPs (~4.0e−9) and for ART applied to GEMMA over 1 kb windows of SNPs (~1.3e−7), respectively. The black dotted line represents the FDR (*α* = 0.10) threshold for independent SNP tests (~7.7e−8). Black circles represent tests of individual SNPs by GEMMA. Orange triangles represent 1 kb windows tested by ART applied to GEMMA results.

**Figure 4:**
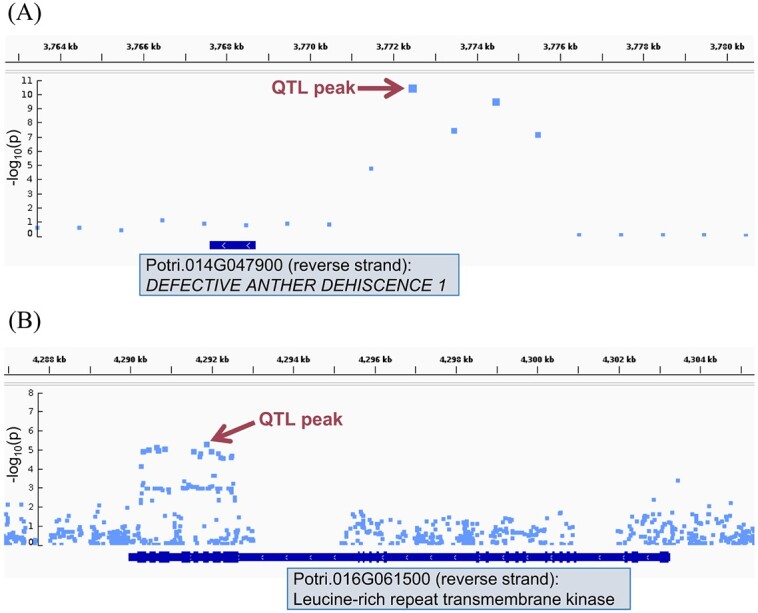
Close-up view of Manhattan plots aligned with *P. trichocarpa* genome annotation (v3.1) using IGV. (A) SKAT results (prior to resampling top associations with MTMC-SKAT) for lateral longest root length (LRL) at Week 3 display an association with a possible promoter region of a putative homolog of *DEFECTIVE ANTHER DEHISCENCE 1.* Here, blue dots represent centers of SNP windows. (B) GEMMA results (without GEMMA-ART displayed) for basal root area at Week 5 display an association with a ~2 kb region of exons and short introns of a putative leucine-rich repeat transmembrane protein kinase. Blue dots represent individual SNPs tested by GEMMA. Purple arrows indicate positions of most significant signal (QTL peaks), as listed in Table 1. Exons are visualized as thickened portions on the gene track. Plots are displayed without MTMC-SKAT and GEMMA-ART *P* values for simplicity; these statistics can be found in Tables S5–S7. Plots were made with IGV and boxes with gene accession IDs, strand of gene and gene information were added manually to IGV plots.

We evaluated results from three distinct GWAS methods (Table 1) to identify candidate QTLs and genes implicated for all 11 traits with *h^2^*_SNP_ > 0.10 [as computed by Genome-wide Efficient Mixed Model Association (GEMMA), Table S1], three of which were evaluated with two different transformations due to ambiguity in the ideal transformation (Tables S1–S3). We term a “QTL peak” as any single-nucleotide polymorphism (SNP) or SNP window associated with a given trait that is not within 30 kb of any other SNP or SNP window with a lower *P* value for the associated trait, thus appearing as the peak position of a group of signals on a Manhattan plot. Only Multi-Threaded Monte Carlo SNP-set (Sequence) Kernel Association Test (MTMC-SKAT) yielded QTL peaks passing the conservative Bonferroni threshold under the assumption that each test (SKAT SNP window) is independent. We tallied the numbers of QTL peaks passing multiple-testing correction thresholds for each method, including the numbers of these QTL peaks within or outside 5 kb of the nearest gene. MTMC-SKAT using 3 kb SNP windows yielded a total of 31 unique Bonferroni-significant associations across all traits. Among these 31 associations, 22 had a window center within 5 kb of an annotated gene. With the stringency of multiple testing relaxed to a threshold for false discovery rate (FDR; *α* = 0.10), a total of 164 unique associations were found to pass this threshold and/or the conservative Bonferroni threshold, and 112 of these had window centers within 5 kb of a gene. The apparent power of SKAT is in contrast to GEMMA and Generalized Mixed Model Association Test (GMMAT); GEMMA yielded only two associations passing the FDR (*α* = 0.10) threshold and none passing the conservative Bonferroni threshold, while GMMAT yielded none passing either. Statistical power for GEMMA and GMMAT was greatly increased by the use of Augmented Rank Truncation (ART) to combine signals across windows of SNPs, which enabled detection of over 100 associations for GEMMA and 8 for GMMAT (Figs 2 and 3). Fig. 4 provides an example of manual inspection of QTL peaks using Integrative Genomics Viewer (IGV) [14]. Associations of note that are addressed in the Discussion are presented in Table 2, with summary statistics for all associations in Table S4 and details presented in Table S5–S8. Gene Ontology (GO) overrepresentation analysis did not reveal any GO terms to be overrepresented among candidate genes.

## Discussion

We identified a large number of putative associations (277) from our GWAS workflow. The use of MTMC-SKAT and ART proved highly valuable in improving statistical power, as 275 of our 277 associations were identified using these methods. We did not find significantly overrepresented GO terms, likely due to the heterogeneity of our candidate genes, which span diverse hormonal pathways and physiological processes.

### Two putative modes of adventitious root regeneration in poplar

**Figure 5:**
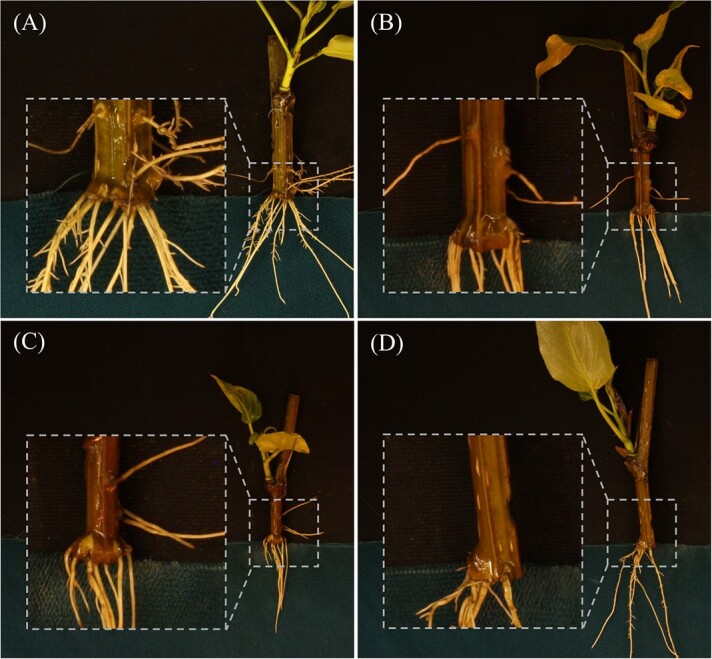
Selected images of root phenotypes highlighting callus or callus-like tissue at the base of stems where basal adventitious roots emerge: (A) genotype BESC-153, (B) BESC-327, (C) GW-9914, and (D) BESC-337.

We observed that when ARs grew from the base of cuttings, the base was often enlarged, distorted, and disorganized in appearance, resembling the calli found in *in vitro* tissue cultures or at *in planta* wound sites (Fig. 5). We term these “basal ARs.” Although we are aware of little research into this specific type of AR in poplar, research in *Populus balsamifera* (a closely related species interfertile with *P. trichocarpa*) suggested that AR development depends on calcium and pH, and that hard callus may inhibit root emergence [15]. More recently, basal ARs growing from callus have been studied in *Pinus* [16]*.* These contrast with ARs growing from the sides of cuttings (“lateral ARs”), which appeared to grow directly from the stem without an intermediate callus stage (Fig. 5). Prior histological research in poplar indicated that lateral ARs appear to originate from secondary meristematic tissue in the cambium [17]. We therefore set up our phenomics pipeline to measure lateral and basal ARs separately, assuming they are biologically distinct and likely originate from distinct lineages of progenitor cells. Their high degree of independence was supported by our PCA analysis, which by inspection of loadings appeared to represent contrasts between the two types of root (Figs S2–S4). Many GWAS associations were for one root type or the other, or for PCs representing contrasts between root types (discussed below).

**Table 1:** Summary of methods for association mapping.

**(A) Method**	**(B) Phenotype input**	**(C) Genotype input**	**(D) Population structure control**
GEMMA [11]	Continuous traits transformed toward normality	~13 million SNPs filtered by 10% missing threshold, 1% MAF threshold	Kinship matrix computed by GEMMA using genotype input described left
GMMAT [12]	Binarized traits		
SKAT [13] with MTMC-SKAT	Untransformed traits	~34 million SNPs filtered by 15% missing threshold and combined into ~0.4 million 3 kb SNP windows (1 kb staggered overlap)	First 6 PCs obtained by PCA over ~10 million SNPs passing 5% MAF threshold

Three GWAS methods (A) were used to study different versions of trait data (B). In addition to enabling the analysis of untransformed traits, SKAT and MTMC-SKAT supported the analysis of rare SNPs and thus a larger genotype dataset (C). Models included controls for population stratification in the form of kinship matrices or PCs from genotype data (D). The same SNP and kinship matrix files were used for GEMMA and GMMAT.

### Phenomics workflow accelerated phenotyping but required human intervention

Our phenomics workflow using computer vision enabled us to extract root trait data from a number of images that would otherwise have been infeasible for humans unassisted. The training of models for this workflow did not require manual preparation of ground-truth semantic labels by humans using an annotation interface but rather utilized ground-truth labels prepared by thresholding and cropping. Although this approach offers the advantage of accelerated training dataset preparation, it lacks the ability for computer vision results to be compared to human-produced ground-truth labels using statistics such as intersection over union. To assess model performance, we inspected each image and grouped them according to notable errors that we observed. The most frequent errors were in accurately segmenting roots from one another and from nonroot tissues or background (Table 3, Fig. S1A and B). Root count statistics were not used in GWAS because of frequent errors and because the summary statistic of aggregate root area provides a proxy for root system proliferation while being more robust to errors. Another common type of error was of incorrectly applied labels, e.g., basal roots labeled as lateral roots or vice versa (Table 3, Fig. S1C). With our system, we attempted to facilitate the labeling of basal and lateral roots by manually placing roots of each type in areas with different background colors (Fig. 1 and Fig. 5 ). We are not aware of other root phenomics workflows that aim to distinguish basal and lateral ARs, although several distinguished the root type for nonadventitious roots, such as for alfalfa [6], *Arabidopsis*, wheat, and *Brassica* [7].

We attempted to correct errors using ImageJ with the SmartRoot plugin (Materials and methods). This error correction was performed on ~18.7% of images. Considering our hybrid approach utilizing both computer vision and manual correction of errant labels, our method is comparable to RootReader2D [8], a tool that similarly performs initial labeling of images and then allows for user correction. The scope of our work did not include the development of a user-friendly and generalizable root annotation method that can be practically applied by other laboratories with diverse imaging conditions and across diverse species of plants. However, several such tools have been recently developed, including RootNav 2.0, which has demonstrated an ability via transfer learning to generalize across different background types and species including wheat, *Arabidopsis*, and *Brassica* [7]*.* The phenomics workflow deployed here is tailored for the specific task of studying poplar root images collected in the manner described. Thus, the system is limited in generalizability and portability. Further work to develop computer visions for root phenomics can provide great value in accelerating studies such as this by prioritizing portability across plants, image types, and diverse root traits of biological interest.

### Candidate genes represent diverse functional roles

#### Regulators of cell division and structure

D-type cyclins regulate the G1-to-S progression of the cell cycle and comprise a family of at least 10 known proteins in *Arabidopsis* and 22 in poplar [18]. Potri.005G141900 encodes a homolog of *CYCLIN D3;2* (*CYCD3;2*), or *CYCLIN D3;3* (*CYCD3;3*) and was previously reported to be differentially expressed across seasonal stages of growth in two *Populus* genotypes with contrasting rates of stem growth and biomass accumulation [19]. In *Arabidopsis*, concurrent loss of function of all three members of the *CYCD3* clade led to retarded seed development, while overexpression of *CYCD3;1* led to premature, irregular, and disorganized division of the hypophysis [20]. Transcription of *CYCD3;3* is negatively regulated by *WUSCHEL-ASSOCIATED HOMEOBOX 5* [21], a poplar homolog of which increases AR number while reducing length in poplar when overexpressed [22]. Although we are unaware of any reports of mutant phenotypes resulting from *CYCD3* overexpression or loss of function in mature roots, several relevant root-related phenotypes have been reported for *CYCD2* and *CYCD4* members. *CYCLIN D4;1* loss of function was reported to reduce basal meristem pericycle cell divisions as well as the number of lateral roots, while these phenotypes were rescued by exogenous auxin, perhaps due to auxin-responsiveness of D-type cyclins with overlapping functional roles [23]. *CYCD2;1* overexpression led to increased root apical meristem cell divisions [24] and increased sensitivity to effects of exogenous auxin in promoting lateral root formation, while loss of function led to reduced auxin sensitivity [25].

Potri.001G149200 encodes a homolog of NOVEL PLANT SNARE 11 (NPSN11), which is believed to interact with other SNARE proteins to provide energy needed for the fusion of membranes that give rise to the cell plate during cytokinesis [26]. Loss of function of *NPSN11* alone yields no apparent mutant phenotype in *Arabidopsis*, putatively due to redundancy with a similar *SNARE* gene. Defects in cytokinesis and embryo development are conferred by simultaneous loss of function of *NPSN11* and the functionally redundant *SNAP33* [27].

Potri.004G210600 and Potri.018G097000 encode members of the *FASCICLIN-LIKE ARABINOGALACTAN* (*FLA*) family, established by gene expression patterns and mutant studies to have vital roles in cell walls and root development [28]. Proteomic analysis of tension wood development in *Populus* provides support for a role of Potri.004G210600 in cell wall structure [29]. An earlier study on *FLA* gene expression in *Populus *examined a subset of *FLA* family members and found many to be preferentially expressed in tension wood [30]. Potri.004G210600 and Potri.018G097000 are annotated based on sequence similarity as putative homologs of *FLA12* and *FLA4,* respectively [31], and a bioinformatic study using sequence alignment and motif analysis corroborated that these are likely homologs, but also indicated that additional *Arabidopsis FLA* genes are also closely related to each of these two candidates and that specific homology is not unambiguous [32]. Research in the *Arabidopsis* model supports a role for *FLA4* in root development related to abscisic acid [33] and for *FLA12* in the vasculature of stems [34]. We are unaware of transgenic studies comparing functions of *FLA4* and *FLA12* side by side during root development or other contexts; further work in this area may help elucidate the extent of functional redundancy or divergence of these and other *FLA* genes within and across tissues.

We report the candidate gene Potri.010G202000 encoding a putative actin-like protein. Cell expansion during root growth is influenced by bundling and rearrangements of actin filaments, regulated at least in part by auxin, cytokinin, and brassinosteroid pathways. *Arabidopsis* mutants of several actin genes display root-related phenotypes including altered root elongation, root morphology, and root hair formation [35].

**Table 2:** Sixteen candidate genes highlighted for discussion and their *Arabidopsis* homologs with putative roles in biological processes of root development.

Candidate genes							Arabidopsis homologs			
Threshold	Trait name	Method	Transformation	Dist.	QTL Position	Accession ID	Description	Accession ID	Score	Similarity
Bonf.	RA PC1	MTMC-SKAT	Untransformed	52	5′	Potri.005G141900	*CYCLIN D3;2; CYCLIN D3;3*	AT 5G67260; AT 3G50070	361; 328	74.9%; 75.9%
Bonf.	RA PC2; basal RA growth; basal RA (wk 5)	MTMC-SKAT	Untransformed	0	Intragenic, nonexonic	Potri.001G149200	*NOVEL PLANT SNARE 11*	AT 2G35190	403	92.3%
FDR (*α* = 0.1)	Overall PC2	MTMC-SKAT	Untransformed	22	3′	Potri.004G210600	*FASCICLIN-LIKE ARABINOGALACTAN-PROTEIN 12*	AT 5G60490	189	83.0%
Bonf.; FDR (*α* = 0.1)	RA PC1; lateral LRL (wk 3)	MTMC-SKAT	Untransformed	913; 0	3′; exonic	Potri.018G097000	*FASCICLIN-LIKE ARABINOGALACTAN-PROTEIN 4*	AT 3G46550	394	82.7%
ART–Bonf.	RA growth	GEMMA	Box–Cox	896	5′	Potri.010G202000	*ACTIN-RELATED PROTEIN 5*	AT 3G12380	1113	87.3%
Bonf.	RA PC2; basal RA (wk 5); basal RA growth	MTMC-SKAT	Untransformed	730; 730; 0	5′; 5′; intragenic, nonexonic	Potri.006G161200	*INDOLEACETIC ACID-INDUCED PROTEIN 16*	AT 3G04730	162	86.0%
Bonf.	Lateral LRL (wk 3)	MTMC-SKAT	Untransformed	3775	5′	Potri.014G047900	*DEFECTIVE ANTHER DEHISCENCE 1*	AT 2G44810	560	88.0%
Bonf.; Bonf.; Bonf.; Bonf.; FDR (*α* = 0.1)	RA PC2; LRL PC2; basal RA growth; basal RA (wk 5); overall PC2	MTMC-SKAT	Untransformed	0	Exonic	Potri.007G056400	*HISTIDINE KINASE 1*	AT 2G17820	1634	81.8%
Bonf.	RA PC1	MTMC-SKAT	Untransformed	10 480	5′	Potri.010G031800	*RUNKEL* (protein kinase family protein with ARM repeat domain)	AT 5G18700	1828	81.7%
Bonf.	RA PC1	MTMC-SKAT	Untransformed	1437	3′	Potri.019G127200	Protein kinase family protein with leucine-rich repeat domain	AT 1G35710	893	68.9%
FDR (*α* = 0.1)	LRL PC2	MTMC-SKAT	Untransformed	4967	5′	Potri.011G028200	*CYSTEINE-RICH RECEPTOR-LIKE PROTEIN KINASE 25*	AT 4G05200	393	65.1%
ART–Bonf.	Basal RA (wk 5)	GEMMA	Box–Cox	0	Exonic	Potri.016G061500	Leucine-rich repeat transmembrane protein kinase	AT 1G53440	622	62.7%
Bonf.	RA PC1	MTMC-SKAT	Untransformed	2296	5′	Potri.015G105000	*PROPYZAMIDE-HYPERSENSITIVE 1* (dual specificity protein phosphatase family protein)	AT 5G23720	1189	81.8%
ART–Bonf.	Lateral LRL (wk 3)	GEMMA	Box–Cox	1165	5′	Potri.001G436800	*GLUTATHIONE S-TRANSFERASE TAU 25*	AT 1G17180	308	80.7%
ART–Bonf.	RA PC2	GEMMA	Outliers removed and RB-INV normal transformation	2242	5′	Potri.004G023100	Peroxidase superfamily protein	AT 3G01190; AT 5G15180	407; 403	75.4%; 75.8%
ART–Bonf.	RA growth	GMMAT	Binarized trait	2675	5′	Potri.008G072700	*FERRITIN2*; *FERRITIN4*	AT 3G11050; AT 2G40300	373; 358	83.1%; 84.9%

For each of these candidates, relevant literature is summarized (Discussion). QTLs were identified from various traits related to root area (RA) and longest root length (LRL), for either basal or lateral ARs, or trends or contrasts across both types. The “threshold” column provides the most conservative significance threshold passed by a given association, among the previously described conservative Bonferroni threshold (Bonf.), FDR (*α* = 0.1), and ART–Bonferroni (ART–Bonf.) methods. The “QTL Position” and “Dist.” columns represent the position and distance of an individual SNP for GEMMA or GMMAT, or the center of a 3 kb SNP window for MTMC-SKAT, in relation to a given candidate gene. Growth traits represent the temporal change in a given trait from Weeks 2 to 5. For candidate genes supported by multiple traits and/or distinct QTL peaks, the traits, significance thresholds, and statistics are given for each and separated by semicolons when they differ. *Arabidopsis* homologs and Smith–Waterman alignments were retrieved from Phytozome (phytozome-next.jgi.doe.gov), and in cases of marked ambiguity, the two top homologs are given and separated by semicolons. These associations and others are detailed in Tables S5–S8, along with information from *P. trichocarpa* gene annotations.

#### Regulators of hormone signaling

Potri.006G161200 encodes a member of the *Aux/IAA* gene family believed to have at least 35 members in *P. trichocarpa* [36]. In *Arabidopsis*, Aux/IAA proteins have long been well characterized as high-level regulators of root development [37]. In *Populus*, at least six transgenic experiments involving the Aux/IAA-interacting TRANSPORT INHIBITOR RESPONSE 1, downstream *AUXIN RESPONSE FACTOR* genes, or their regulators have demonstrated important roles of Aux/IAA-dependent pathways in AR development [3]. Moreover, the role of these pathways in rooting likely depends in part on crosstalk with ethylene and JA pathways [38], transgenic alteration of which also alters AR development in *Populus* [3].

Further evidence for a role of JA signaling in rooting of poplar is indicated by an association with Potri.014G047900, encoding a homolog of DEFECTIVE IN ANTHER DEHISCHENCE 1 (DAD1), which catalyzes a key step of JA biosynthesis [39]. Our previous GWAS of *in planta* regeneration in poplar supports a major role for JA signaling in callus and shoot regeneration [40], via pathways that are likely to be relevant to AR considering the previously discussed emergence of basal ARs from callus.

#### Regulators of post-translational modifications

Among our candidate genes, we report a notable number of genes encoding putative catalysts of PTMs, including a histidine kinase (Potri.007G056400), serine/threonine kinase (Potri.010G031800), tyrosine kinase (Potri.019G127200), dual-specificity kinases (Potri.011G028200, Potri.016G061500), a dual-specificity phosphatase (Potri.015G105000), and a glutathione-*S*-transferase (GST, Potri.001G436800). Protein function was inferred from annotated functional domains of *Arabidopsis* homologs from The *Arabidopsis* Information Resource [41]. We are unsure of the precise mechanisms by which these candidates affect root traits because of the wide prevalence of PTMs in eukaryotes. Evidence has been found for PTMs of over 12 000 substrates in *Arabidopsis* [42], likely spanning all pathways implicated by our candidate genes.

#### Regulators of reactive oxygen species signaling

ROS can affect or correspond to root development through mechanisms involving oxidative stress, activation or deactivation of proteins including transcription factors [43] and hormone metabolism [44], among others. The putative GST encoded by Potri.001G436800 may influence AR via the antioxidant properties of glutathione if not through other roles, such as those involving PTMs as discussed above. *Arabidopsis* mutants of a GST family gene display phenotypes including altered root growth and branching, and relative insensitivity to abiotic stress or abscisic acid treatment [45]. Potri.004G023100 is an example of a putative peroxidase that may also regulate redox balance. Oxidative stress is mitigated in part by ferritins, proteins that sequester Fe and thus prevent Fe from reacting with oxygen and producing oxygen radicals. Potri.008G072700 encodes a homolog of the four-member ferritin family in *Arabidopsis* and is most closely related to *FERRITIN 2* (greater Smith–Waterman alignment score) and *FERRITIN 4* (greater residue similarity, Table 1). The latter homolog has been studied in the context of root system architecture. Addition of chelated Fe in media leads to an increase in lateral root density, which is disrupted with triple loss of function of *FERRITIN 1*, *3*, and *4* [46].

**Table 3:** Descriptions of error types and their frequencies.

Error ID	Description	Frequency among images
0	No significant error	81.3%
1	Failure to segment some or all roots	8.7%
1A	Failure to segment all roots	1.3%
1B	Failure to segment basal root despite segmenting lateral root	0.2%
2	Incorrectly identified feature (e.g., basal root labeled as lateral)	4.6%
3	Root label shorter than root in image (truncation)	2.9%
4	Errant detection of root where no root exists	0.3%
5	Other error or combination of errors	2.2%

The most common errors involved failure to segment whole roots (Error ID 1, including Subtypes 1A and 1B as well as additional uncategorized issues) and mislabeling of basal or lateral roots (Error ID 2). In addition, many errant annotations featured truncated segments (Error ID 3) or complex mixtures of error types (Error ID 5).

### Agreement with previous association mapping in poplar

We cross-referenced our candidate genes with those found in four association mapping studies of rooting or other developmental traits in poplar, including QTL interval mapping of AR and shoot traits in *Populus deltoides × simonii* [5], and GWAS of diverse wood, growth, and seasonal traits in *P. trichocarpa* [47–49]. Very few of our candidate genes were previously identified by QTL interval mapping as possible regulators of AR traits. These include an uncharacterized gene believed to be a transcription factor (Potri.005G154200), as well as a putative xyloglucan endotransglucosylase/hydrolase (Potri.016G098600), both found as an association for lateral longest root length (LRL) at Week 3, and in this prior work for total root number. An uncharacterized putative phospholipase (Potri.015G026500) was also found as an association with LRL at Week 3 in our work, and in this prior work with root volume and total root number, as well as two shoot-related traits. Finally, we found a putative heme-binding protein (Potri.016G098500) associated with our first PC of LRL traits (across root types and time points) and previously found as an association with LRL. Possible reasons for the relatively low level of overlap between these studies include differences in gene diversity and physiology across species of poplar, variation in association mapping characteristics and statistical methods, and the use of different rooting assays [5]. We also found 11 of our candidate genes to overlap with those found from poplar association mapping of various developmental traits for nonroot tissues. Of note, a putative homolog of the transcription factor *WKRY13* (Potri.005G086400) was found as an association with bud set and leaf drop [49], as well as our measure of total root growth (Weeks 2–5). *WRKY13* is likely to have roles across diverse developmental contexts including stress response [50], stem development [51], and flowering [52], and we are unaware of evidence indicating whether roles in bud and root development are related via particular transcriptional targets of WRKY13. A likely homolog of the transcription factor *ABSCISIC ACID-INSENSITIVE 5* (*ABI5*, Potri.006G083000) was similarly found as an association for bud set [49] as well as our PC1 of root development. *ABI5* roles in flowering may depend largely on transcriptional regulation of the downstream *FLOWERING LOCUS C* [53], while significance for root development may be a consequence of roles in stem cell maintenance [54], phytohormone crosstalk [55], and/or nitrate response [56]. Statistics and annotations for these associations and nine others overlapping across our work here and prior studies [5, 47–49] are given in Tables S5–S7.

## Conclusion

We performed GWAS to identify regulators of AR capacity in 1148 genotypes from a *P. trichocarpa* clone bank. To facilitate the collection of quantitative measures of adventitious root development, we employed a phenotyping system tailored for our AR assay in poplar. The hundreds of candidate genes identified include regulators of cell division and structure, hormone signaling, ROS signaling, and PTMs as well as many genes of miscellaneous or unknown function. The distinct origins of basal and lateral roots were supported both by our multivariate phenotype analysis and GWAS associations. As root development is a complex and polygenic process, future research will benefit from functional studies such as through mutagenesis, *in silico* statistical tests for epistasis, *in vitro* assays to identify and confirm possible gene–gene or gene–protein interactions, and differential rooting responses to environmental treatments.

## Materials and methods

### Plant materials

We used clones from a *P. trichocarpa* GWAS population that was recently expanded to include a total of 1323 genotypes [40, 57]. This population represents variation in wild *P. trichocarpa* spanning regions of British Columbia, Washington, Oregon, Idaho, and Northern California. Clone banks for this GWAS population were produced in multiple locations, among which a replicate in a Corvallis, OR, field location was utilized to obtain cuttings for this study. In an effort to study as many genotypes as possible given limited time and contamination of some cuttings, we studied a total of 1148 genotypes from this population. Each genotype was studied in replicates of two, as maximizing population size was prioritized over clonal replication and can be expected to provide similar benefits in reducing statistical noise. This study was performed using materials collected as described in previous work [40]. Cuttings of ~2 cm in diameter were collected from actively growing branches near the middle of small trees 2–5 m in height; for consistency, we sought to collect cuttings from similar positions on each tree. Collections were performed in the winters of 2018 and 2019 (Table S9). Cuttings were stored at −10°C for timespans listed in Table S9, and then used for rooting assays.

### Assay of rooting

Cuttings were placed in 50 mL Falcon tubes with tap water and allowed to root. The water contained no rooting hormones or other additives. As water evaporated, the tubes were refilled with fresh tap water every 3–4 days. No fungal growth was observed in the water. Beginning 2 weeks after cuttings were first placed in water, images were collected at weekly time points for 4 weeks. Prior to taking each image, plants were removed from water and placed on top of a surface with roots arranged to separate putative lateral and basal roots. To aid their recognition by our computer vision pipeline, basal roots were laid downward atop blue felt while lateral roots were laid to the side on gray felt. Each image also included a label and ruler. Plants were imaged from above using a Canon Rebel XSi DSLR camera attached to a mount and facing downward. Due to practical limitations in the number of cuttings that could be studied at once, the study was divided into eight “batches,” each of which featured up to 400 cuttings, including 2 replicates for each of up to 200 given genotypes (Table S9).

### Computer vision pipeline

We adopted the DeepLab network [58] with backbone ResNet50 [59] in our segmentation models because of the efficiency and accuracy of this architecture. We trained two different segmentation models: the first was used to segment an image into background, plant, ruler, and label (Deep Model 1); the second was used to segment the plant into leaf, stem, and root (Deep Model 2). Below, we introduce how we collected training labels to train the two networks and then used the two networks to measure biological traits of interest.

Due to variations in camera settings between “portrait” and “landscape” modes, and a desire to avoid artifacts resulting from this inconsistency, we first rotated images to a uniformly landscape orientation. Next, the background was segmented based on color thresholding. As the plant, ruler, and label varied in color and were found in approximately similar positions from image to image, we first segmented these components based on their spatial positioning and colors using mean-shift segmentation and *k*-means clustering. Images successfully segmented as such were used to produce a training set with ~500 images used to train a deep model for segmentation. Inference was performed using this model, and correct examples were used to retrain the model with an expanded dataset, resulting in a final model trained with 2239 examples (Deep Model 1). Afterward, to segment the plant into stem, root, and leaf, we performed mean-shift segmentation and applied a location threshold to produce a training set of ~900 images. These were used to train a second deep model, and the training set was again expanded by running inference on new images and selecting correct results, resulting in a final training set with 3496 training examples (Deep Model 2).

Following training of both deep segmentation models, they were applied for inference of the full dataset. All images were standardized to the same orientation, followed by deployment of both models. The final result of segmentation was separation of background, leaf, stem, root, ruler, and label for each image. Next, the segmentation results were further analyzed to produce statistics on biological traits of interest. Since the camera height varied across images, we computed the number of pixels per ruler width for each image to enable standardization via the actual size represented by a single pixel.

We proceeded to compute the lengths and areas of roots on the metric scale, as well as the diameters of stems. Root statistics were computed as follows. (1) First, we further segmented roots and attempted to label individual roots by finding distinct connections to the stem or root of origin (for branch roots) and finding the longest paths of root pixels from these origins to distinguish major roots from immature branch roots and root hairs. (2) The background was classified as top background and bottom background based on the color of the felt background underneath the plant, allowing each root to be classified as lateral or basal depending on the background. (3) For each basal and lateral root, we computed root length by computing the shortest path from root tip to origin and root area by counting root pixels. LRL and total root area were computed separately for basal and lateral roots in each image. (4) All statistics were converted from pixel scale to the metric scale using estimates of pixel size obtained from the ruler in each image. RGB and false-color images for key steps of the segmentation process are shown in Fig. 1, which presents key steps of the imaging workflow.

### Phenotype error evaluation

Source images were compared to segmented images (Fig. 1) to identify cases where data contained errors for the length, area, type, or number of roots. During review of images, a list of types of common errors was built. Each image was reviewed and scored on a spreadsheet according to the error or errors observed, if any (Table 3). Images were then sorted into folders according to the types of data errors they contained using this spreadsheet and R scripting. Finally, the below methods were applied to obtain corrected measurements for images with errors, and initial measurements were overwritten with these corrections.

### Phenotype correction

ImageJ version 1.53a software and the SmartRoot version 4.21 plugin [60] were used to make all measurements for corrections. Original RGB images collected by the camera were opened in ImageJ, then converted to 8-bit grayscale, and inverted to support visualization (with light roots on dark background). Roots that required correction of length data were then traced using the SmartRoot Trace Root tool. To calculate root areas, an image brightness threshold was adjusted until only the roots were highlighted, then the area was selected and measured using the ImageJ Wand tool. Root types were corrected based on manual judgment of whether a root was a lateral root or basal root, while recalling that these were placed on different areas of felt during imaging as previously described.

### Phenotype preparation for association mapping

We obtained a total of 24 traits directly from the computer vision workflow. These include four time points each for three types of root area traits (lateral root area, basal root area, total root area) and three types of LRL traits (lateral LRL, basal LRL, overall LRL; Table S2). For each of these traits, mean values of each trait were computed across two replicates for each of 1148 genotypes and used for downstream GWAS. PCA was performed using ‘stats::prcomp’ in R [61] to produce PCs representing trends across traits and time points. PCA was performed over three groups of traits: (1) area of basal or lateral roots at all four time points; (2) LRL for basal or lateral roots at all four time points; and (3) all basal or lateral root traits, including area and LRL of basal or lateral root at all four time points. Scree plots were consulted to determine the numbers of PCs that represent significant variation for each group of traits over which PCA was performed, and two significant PCs from each group were thus used for downstream GWAS (Figs S2–S4). We also computed three “growth” traits representing the difference in lateral, basal, or total root area between the first (Week 2) and last (Week 5) time points.

Normality of traits was assessed in parallel with traits from our GWAS of *in planta* regeneration in poplar, using the same methods [40]. In short, we assessed normality of untransformed traits using Q–Q plots, histograms, Shapiro–Wilk tests, and Pearson correlation coefficients with theoretical normal distributions, then applied necessary transformation and other treatments including Box–Cox transformations, removal of zero values, and removal of outliers on a case-by-case basis for conservative but adequate transformation of each trait (Tables S2–S3). Transformations were applied for all traits and considered adequate when histograms and Q–Q plots indicated that transformed distributions were approximately normal (Fig. S5). Since results from Box–Cox transformation were notably flawed for PC traits, we performed downstream analysis using two versions of each PC trait tested, including one transformed via Box–Cox and another transformed by a rank-based inverse (RB-INV) transformation (Table S3).

### Association mapping

Association mapping of all traits was performed in parallel with traits in our published GWAS of *in planta* regeneration, using methods and SNP sets detailed in this previously reported work [40]. To summarize, three GWAS methods were used: (1) GEMMA [11]; (2) GMMAT [12]; and (3) SKAT [13] with the MTMC-SKAT R extension we developed. Versions of trait data transformed toward normal distributions were analyzed with GEMMA, while binarized traits were analyzed with GMMAT and untransformed traits with MTMC-SKAT using resampling to avoid violations of linear model assumptions for high-confidence associations. This work utilized a previously described SNP set featuring a total of 40.4 million SNPs subsequent to initial quality filtering [40] and prior to the filtering steps we describe here. GEMMA and GMMAT were run using kinship matrices to adjust for population stratification, computed by GEMMA using ~13 million SNPs with a minor allele frequency (MAF) threshold of 1% and that are missing in no more than 10% of genotypes. As an aside, the number of SNPs analyzed by GEMMA was further reduced to ~12.8 million during analysis because GEMMA performed additional filtering with a default *R*^2^ threshold of 0.9999 (correlation between SNPs and covariates) and MAF and missing rates of 0.01 and 0.10, which were recomputed at this stage. GEMMA also provided a measure of the percentage of trait variance explained by additive genetic effects (represented by the kinship matrix) under the null model, which we consulted as a type of narrow-sense SNP heritability (*h*^2^_SNP_). MTMC-SKAT was run with covariates of six principal components derived from common SNPs (MAF > 0.05) to correct for population stratification, and explanatory variables from a set of ~34.0 million SNPs with missing rate below 15% and including rare SNPs (low MAF). Up to 10 million permutations were performed to compute a given empirical *P* value, which could thus be resolved to values as low as 1e−7. MTMC-SKAT was deployed on the high-performance cluster COMET, made available through NSF XSEDE [62]. Stem diameter and batch were included as covariates for all GWAS methods. PLINK [63] was used for SNP filtering with aforementioned parameters for each SNP set. SNPs that were not mapped to chromosome scaffolds were excluded prior (for MTMC-SKAT) or subsequent to association mapping (for GEMMA/GMMAT). These methods are summarized in Table 2.

To identify QTLs from results that are statistically significant, we computed multiple testing correction thresholds using the Bonferroni method (parameters: *α* = 0.05, *N* tests equal to *N* SNPs for GEMMA/GMMAT or *N* SNP windows for SKAT) and Benjamini–Hochberg FDR (*α* = 0.10) via ‘stats::p.adjust’ in R [61]. We further sought to identify candidate genes that failed to meet significance according to either of these criteria but were represented by a peak of QTLs showing a pattern of linkage disequilibrium (LD) decay suggestive of a causative association. Toward this end, we applied an implementation of the ART [64] over GEMMA and GMMAT results as we previously described [40]. To summarize, using ART, Wald *P* values from GEMMA or GMMAT were grouped into 1 kb windows, followed by computation of a test statistic and *P* value for their combined effect. A Bonferroni threshold for ART results was computed (*N* tests equal to *N* 1 kb ART windows), henceforth “ART–Bonferroni” and is to be distinguished from the previously described Bonferroni threshold (*N* tests equal to *N* SNPs), henceforth “conservative Bonferroni.” To identify genes near to or encompassing QTLs, we used R scripts with InterMineR [65] to retrieve and parse annotation data from PhytoMine for v3.1 of the *P. trichocarpa* genome [31]. In addition, we inspected candidate genes using zoomed-in Manhattan plots aligned with genome annotations in IGV 2.0 [14].

We consulted the *P. trichocarpa* genome annotation [31] for relatively high-confidence *Arabidopsis* homologs of candidate genes and performed GO overrepresentation analysis of the *Arabidopsis* homologs across biological process, molecular function, and cellular component GO categories via Fisher's exact tests with PANTHER [66, 67]. This GO analysis was performed for genes within 5 kb of QTL peaks across three groups of traits, including subsets of traits for basal or lateral AR development, and for all root traits. GO overrepresentation results were filtered with an FDR correction (*α* = 0.05).

## Acknowledgements

We thank the National Science Foundation Plant Genome Research Program for support (IOS #1546900, Analysis of genes affecting plant regeneration and transformation in poplar), and members of GREAT TREES Research Cooperative at OSU for its support of the Strauss laboratory. Support for the Poplar GWAS dataset is provided by the U.S. Department of Energy, Office of Science Biological and Environmental Research via the Center for Bioenergy Innovation (CBI) under Contract No. DE-PS02-06ER64304. The Poplar GWAS Project used resources of the Oak Ridge Leadership Computing Facility and the Compute and Data Environment for Science at Oak Ridge National Laboratory, which is supported by the Office of Science of the US Department of Energy under Contract No. DE-AC05-00OR22725. We would like to thank the efforts of the personnel from the CBI in establishing the GWAS resource used for this study. This work used the COMET high-performance cluster at the San Diego Supercomputing Center (University of California, San Diego) made available through the Extreme Science and Engineering Discovery Environment (XSEDE), which is supported by National Science Foundation grant number ACI-1548562.

## Author Contributions

Strauss, Fuxin, Jiang, and Muchero designed and directed the overall study and obtained funding for its execution; Ma and Peremyslova designed and/or executed the phenotypic analyses; Nagle, Yuan, and Kaur created, adapted, and executed the computer vision, computation, and data analysis pipelines; Zahl and Peremyslova performed manual inspection and correction of phenotype data; Nagle investigated the candidate genes; Niño de Rivera assisted with inspecting the results in IGV; Nagle wrote the manuscript with editing from Strauss; and all others contributed further edits and revisions.

## Data Availability

Raw data and the code used for this project are available upon request to the authors. GitHub repositories are online for MTMC-SKAT (https://github.com/naglemi/mtmcskat) and the computer vision workflow (https://github.com/jia2lin3yuan1/GWAS-Root-analysis).

## Conflicts of Interest statement

None declared.

## Supplementary Data

Supplementary data is available at *Horticulture Research* online.

## Supplementary Material

Web_Material_uhad125
