# Supplementary material for: GWAS identifies candidate genes controlling adventitious rooting in Populus trichocarpa
Source: Hortic Res. 2023 Jun 14;10(8):uhad125. doi: 10.1093/hr/uhad125 (PMC10407606; doi:10.1093/hr/uhad125)
Supplement: Web_Material_uhad125 [file web_material_uhad125.zip › Fig_S1-S5.docx]

(A)


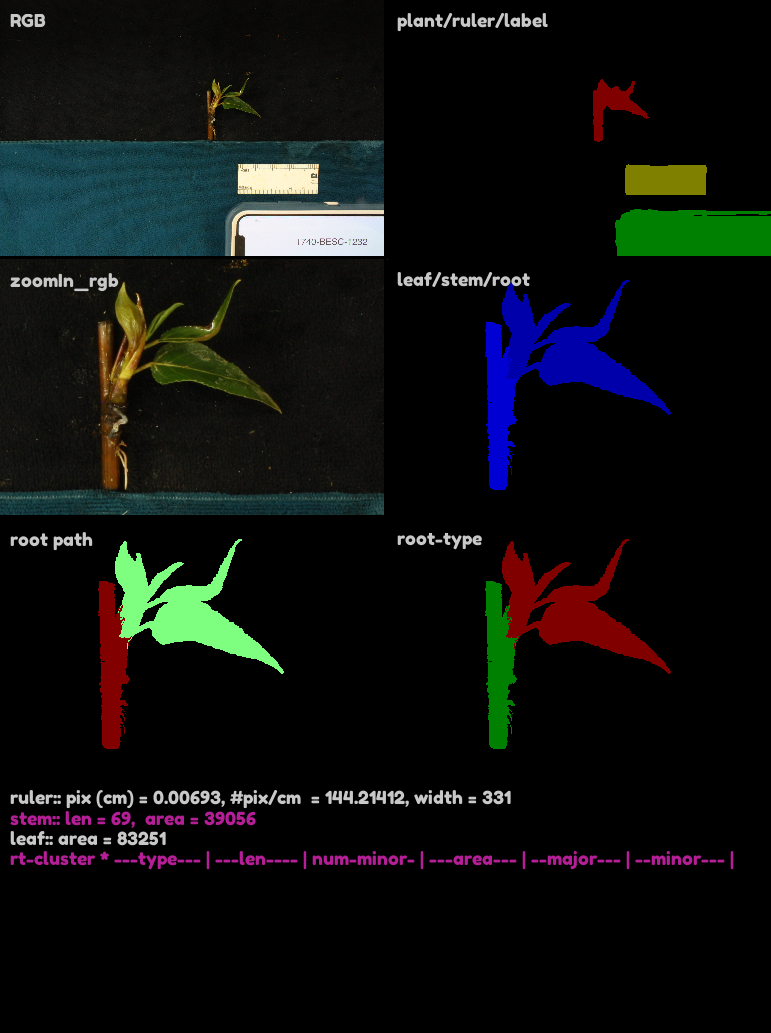


(B)


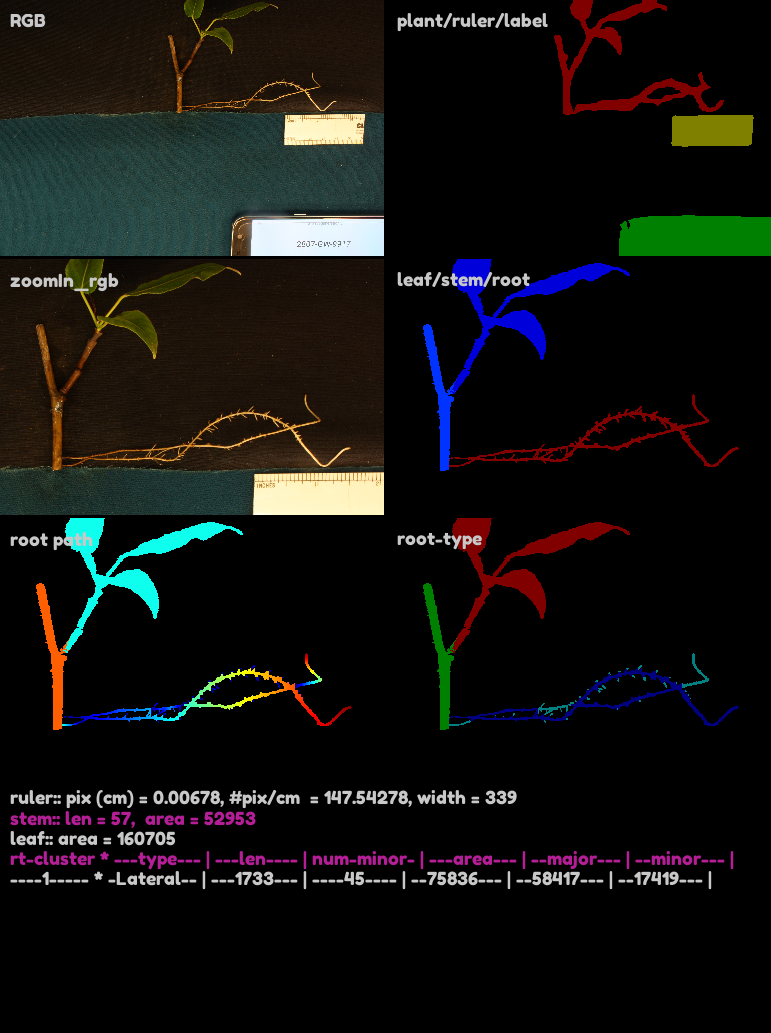


(C)


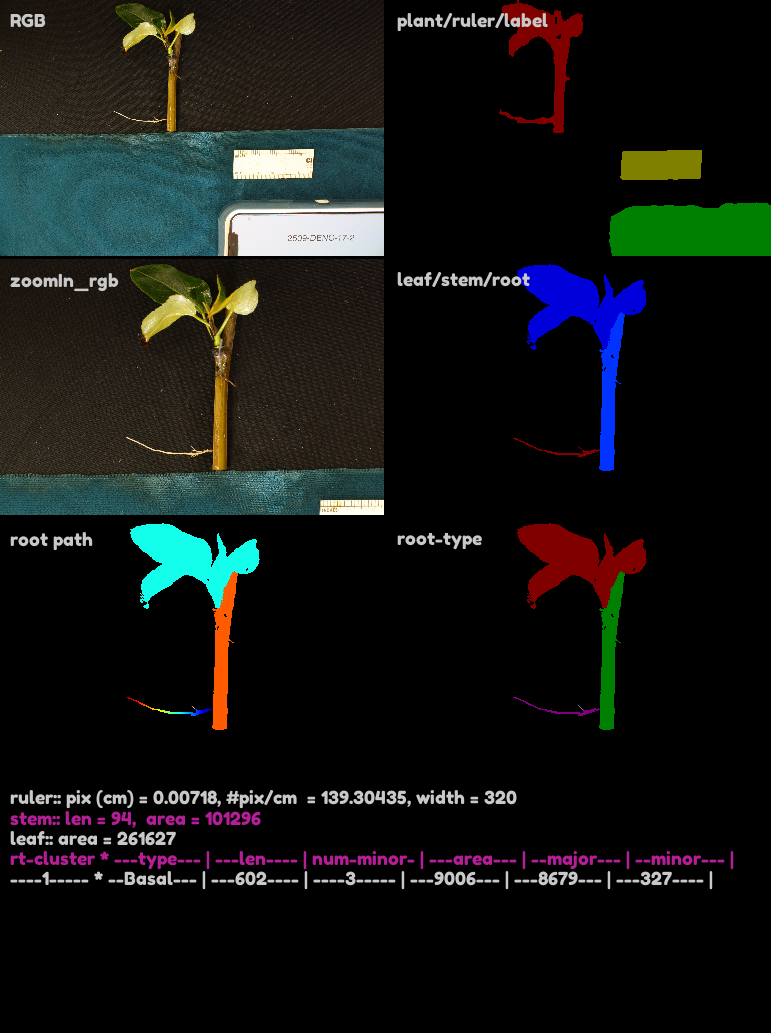


(D)


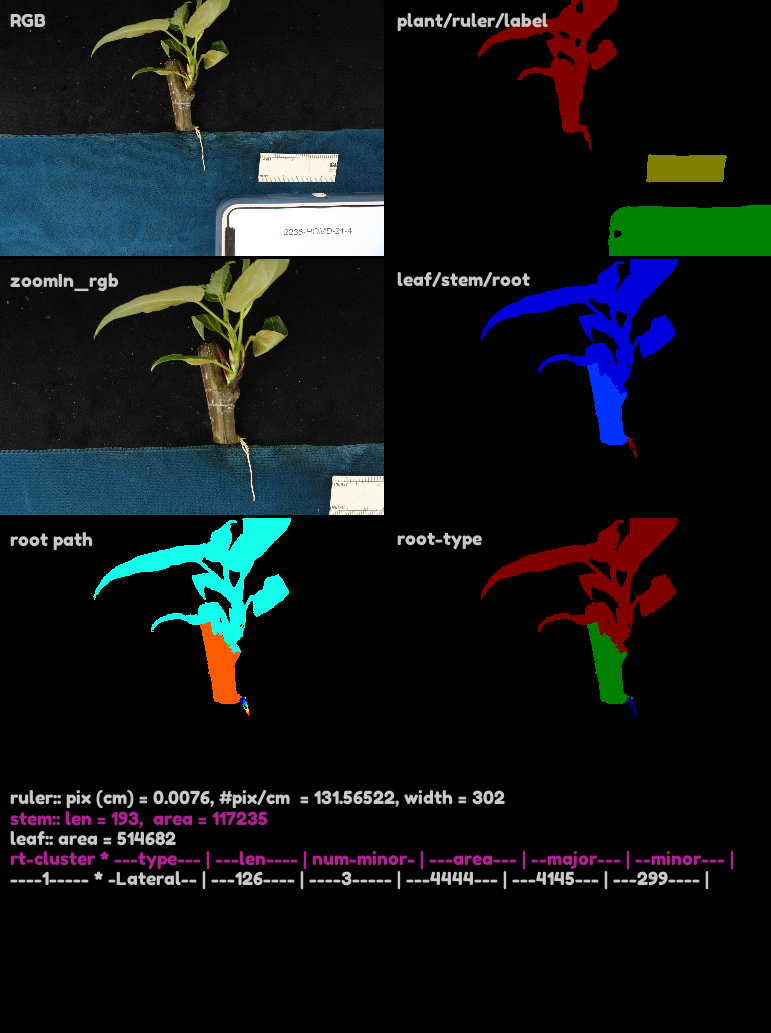


**Supplemental Figure 1.** Examples of common errors in computer vision workflow: **(A)** Example of failure to segment root, in which a root is errantly labeled as background. **(B)** Another type of failure to segment root, in which two roots are errantly labeled as a single root. **(C)** Example of a lateral root misclassified as a basal root. **(D)** Example of a truncated root segment, leading to an errantly short measure of root length. For each example, RGB image data is shown (left) alongside a segmentation map (right) in which root is labeled red and other plant material is labeled blue (with stem and leaf assigned distinct shades of blue).


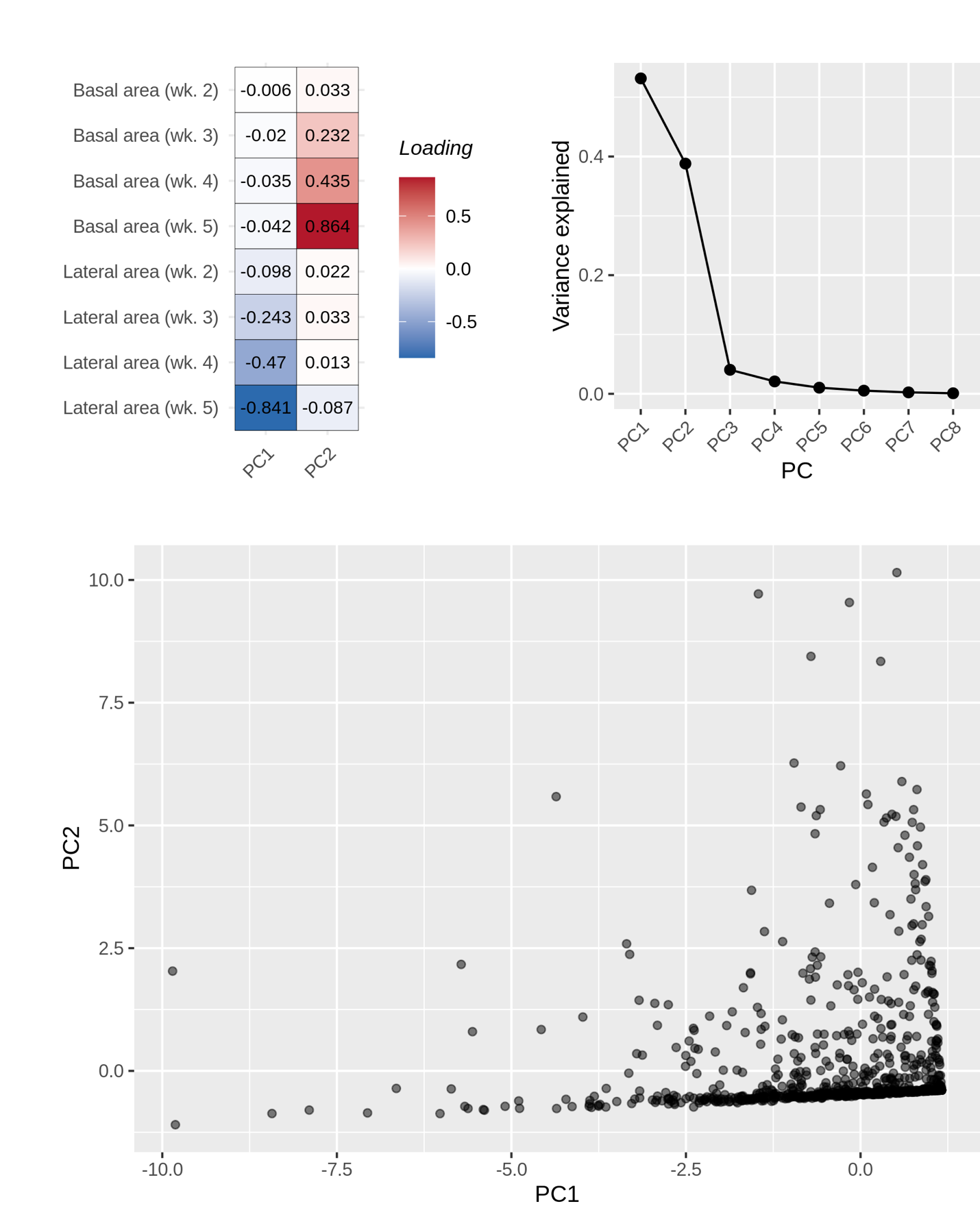


1. (B)

(C)

**Supplemental Figure 2.** Results from PCA over root area traits, across root type (basal or lateral) and all four timepoints of data collection: **(A)** Loadings for top two PCs; **(B)** Scree plot showing proportion of variance explained by each PC; **(C)** PC1 vs. PC2 scatter plot.


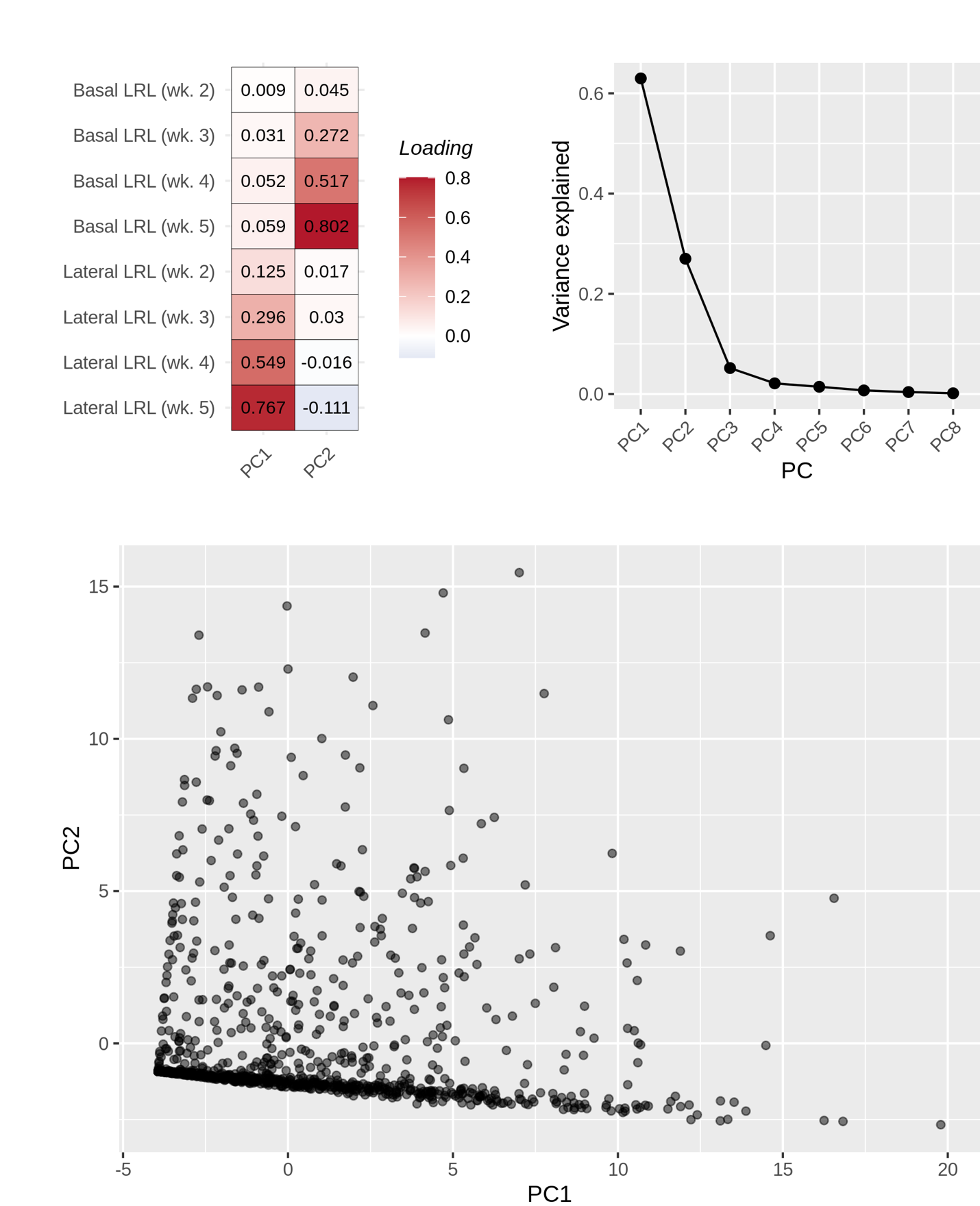


1. (B)

(C)

**Supplemental Figure 3.** Results from PCA over longest root length (LRL) traits, across root type (basal or lateral) and all four timepoints of data collection: **(A)** Loadings for top two PCs; **(B)** Scree plot showing proportion of variance explained by each PC; **(C)** PC1 vs. PC2 scatter plot.


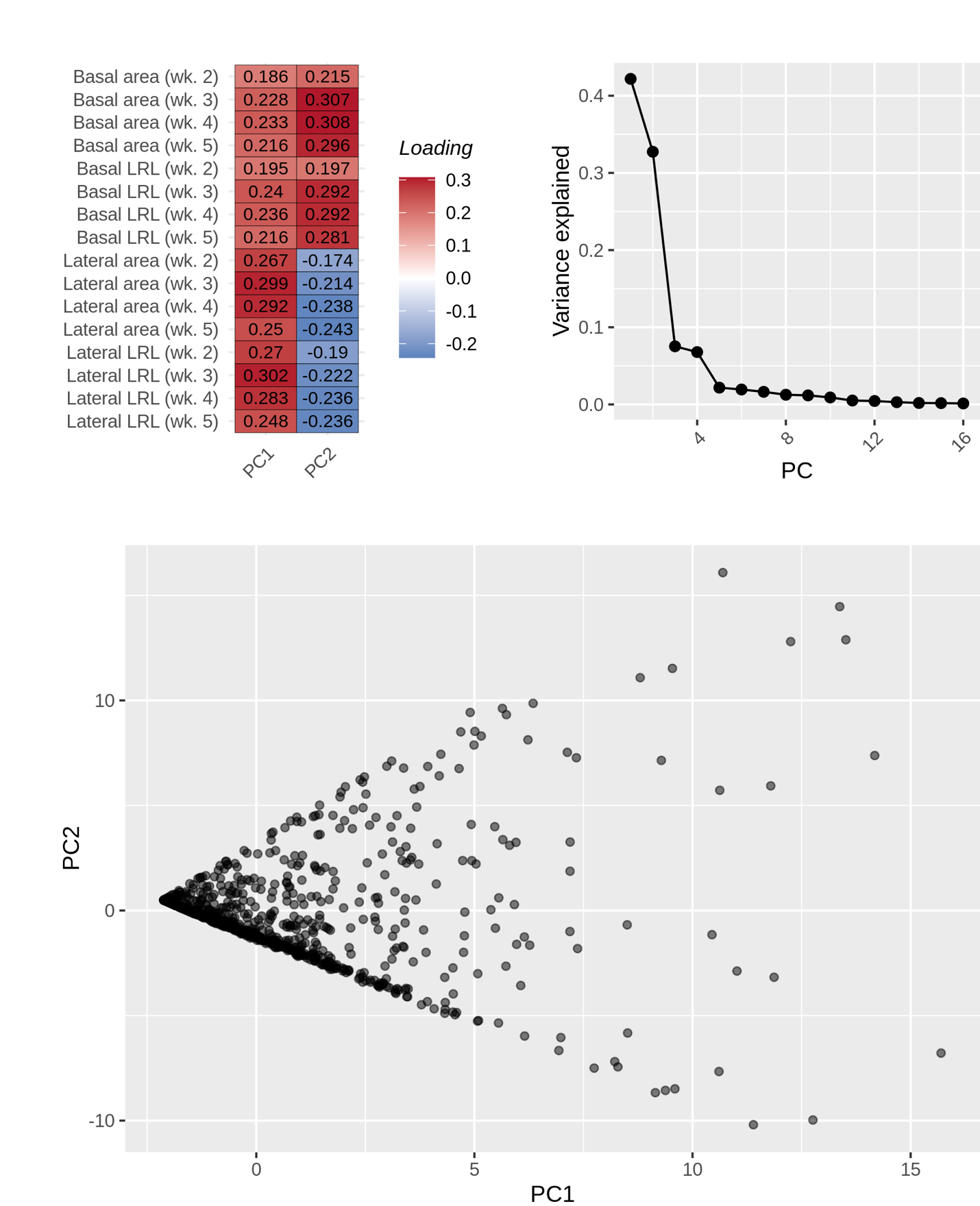


1. (B)

(C)

**Supplemental Figure 4.** Results from PCA over longest root length (LRL) and root area traits, across root type (basal or lateral) and all four timepoints of data collection: **(A)** Loadings for top two PCs; **(B)** Scree plot showing proportion of variance explained by each PC; **(C)** PC1 vs. PC2 scatter plot.


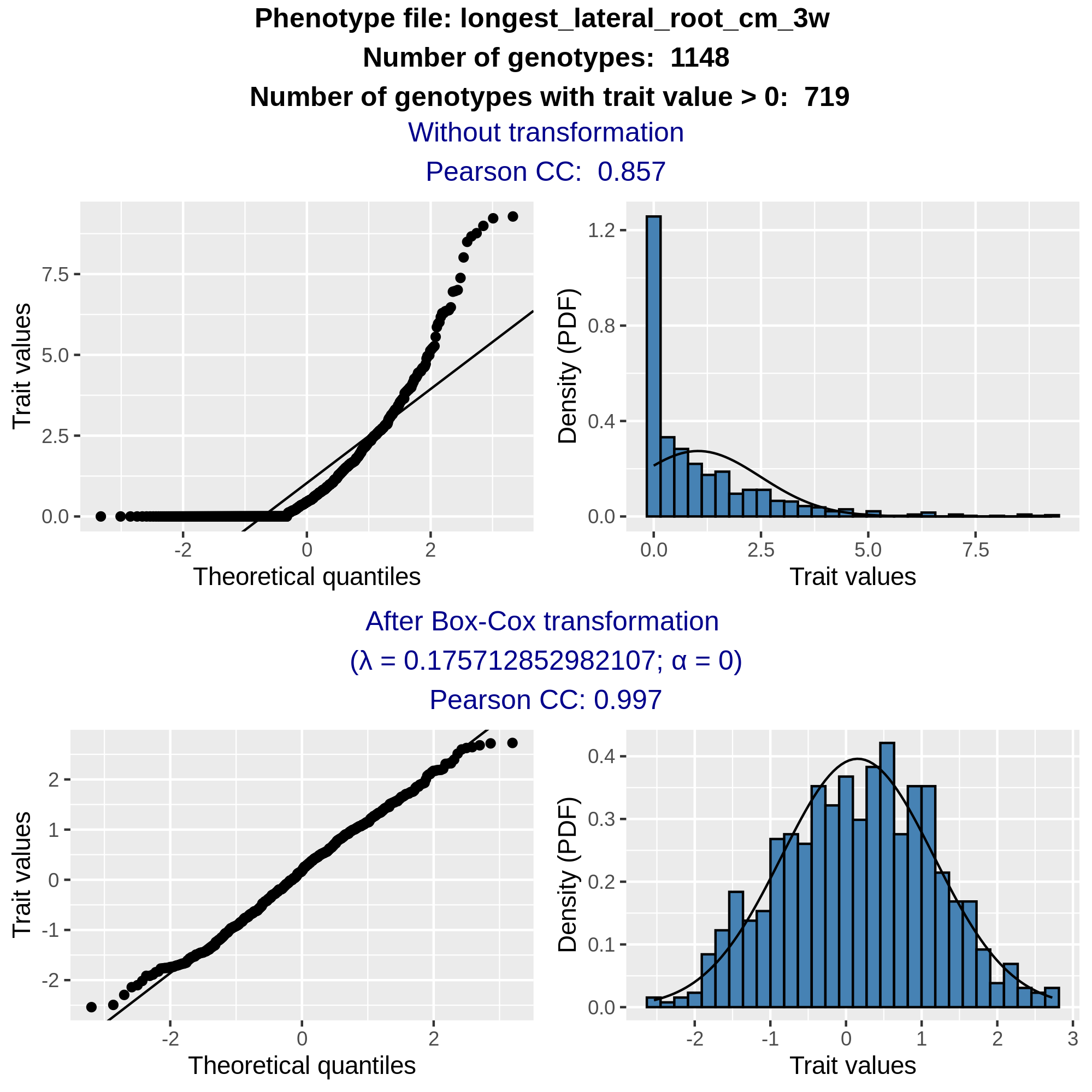

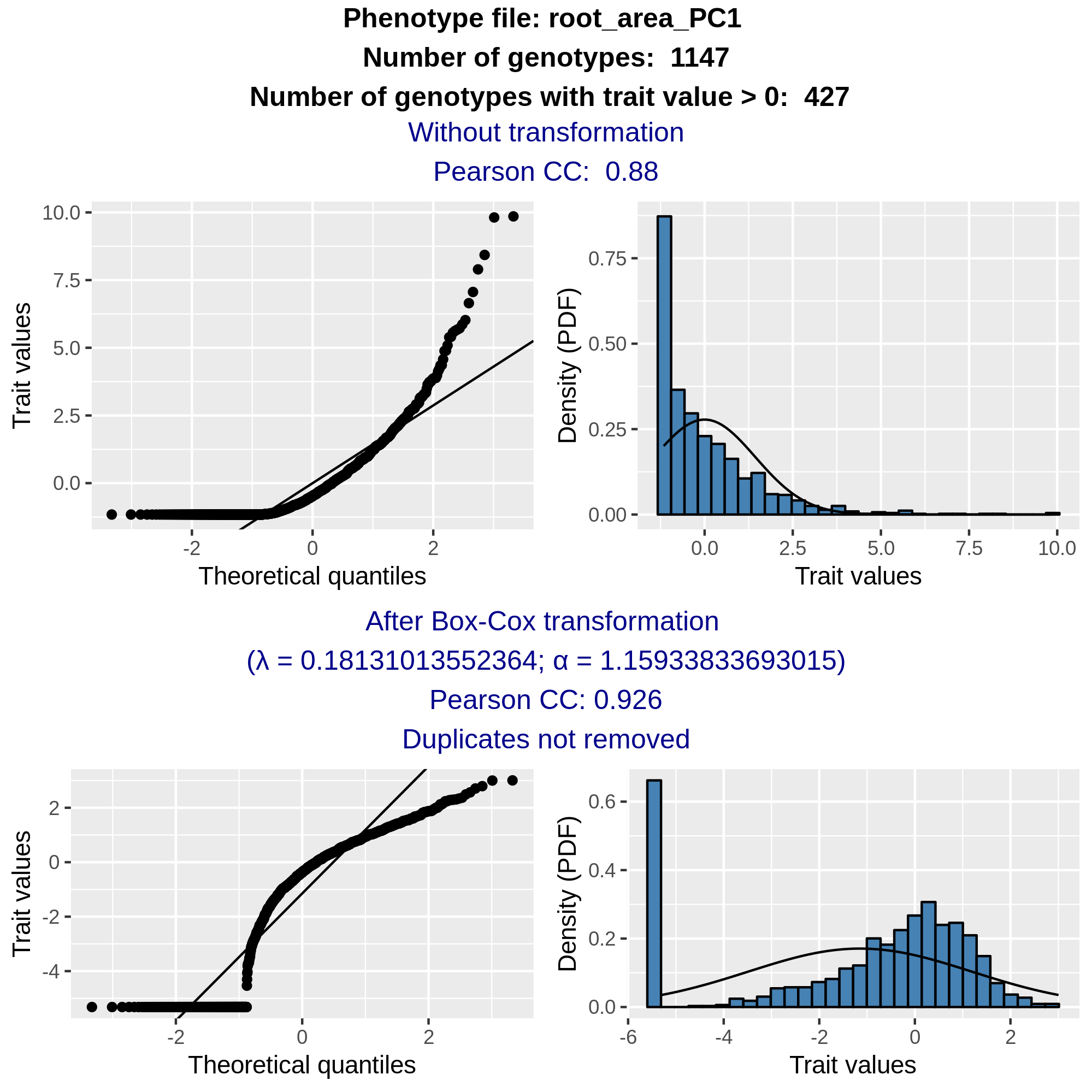

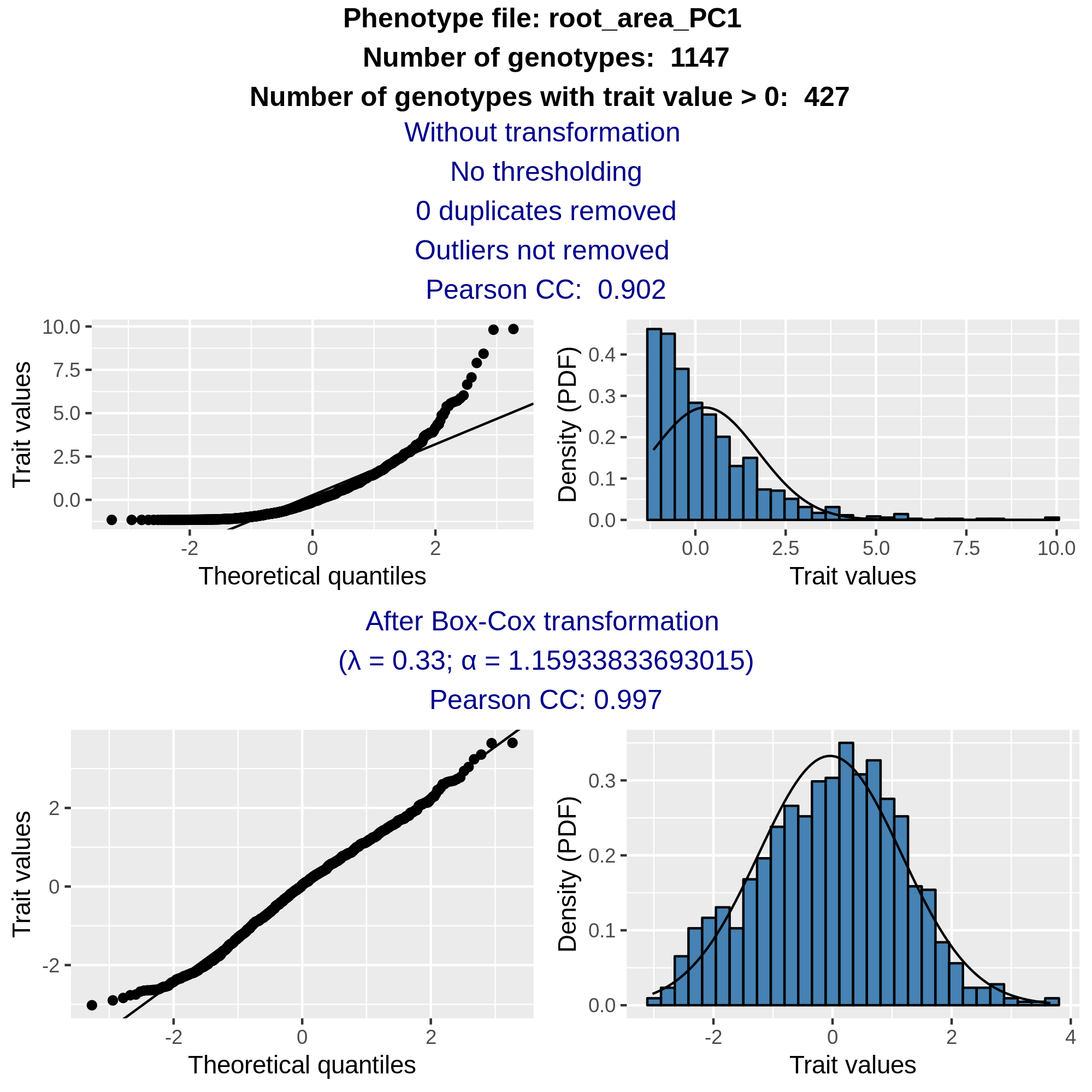

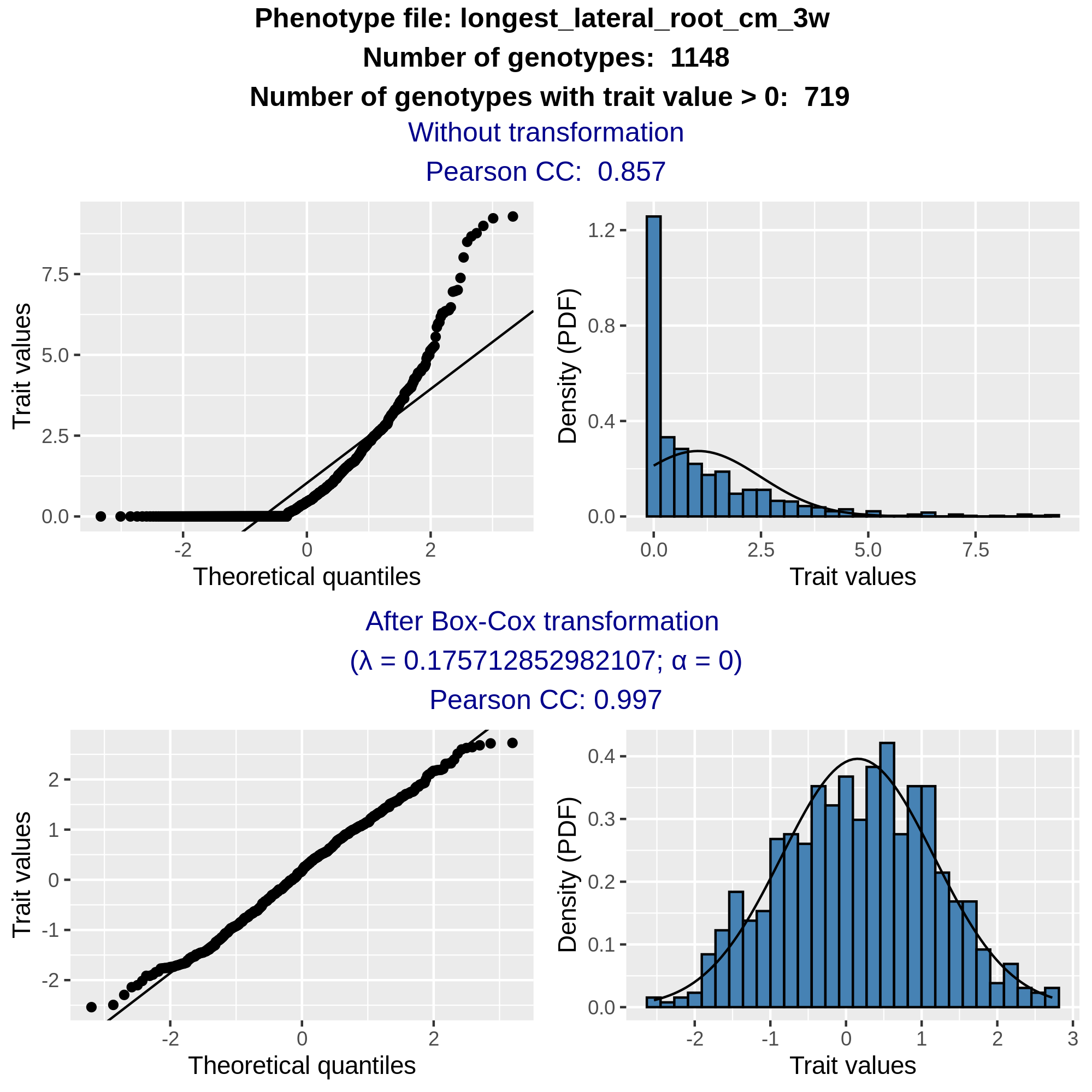

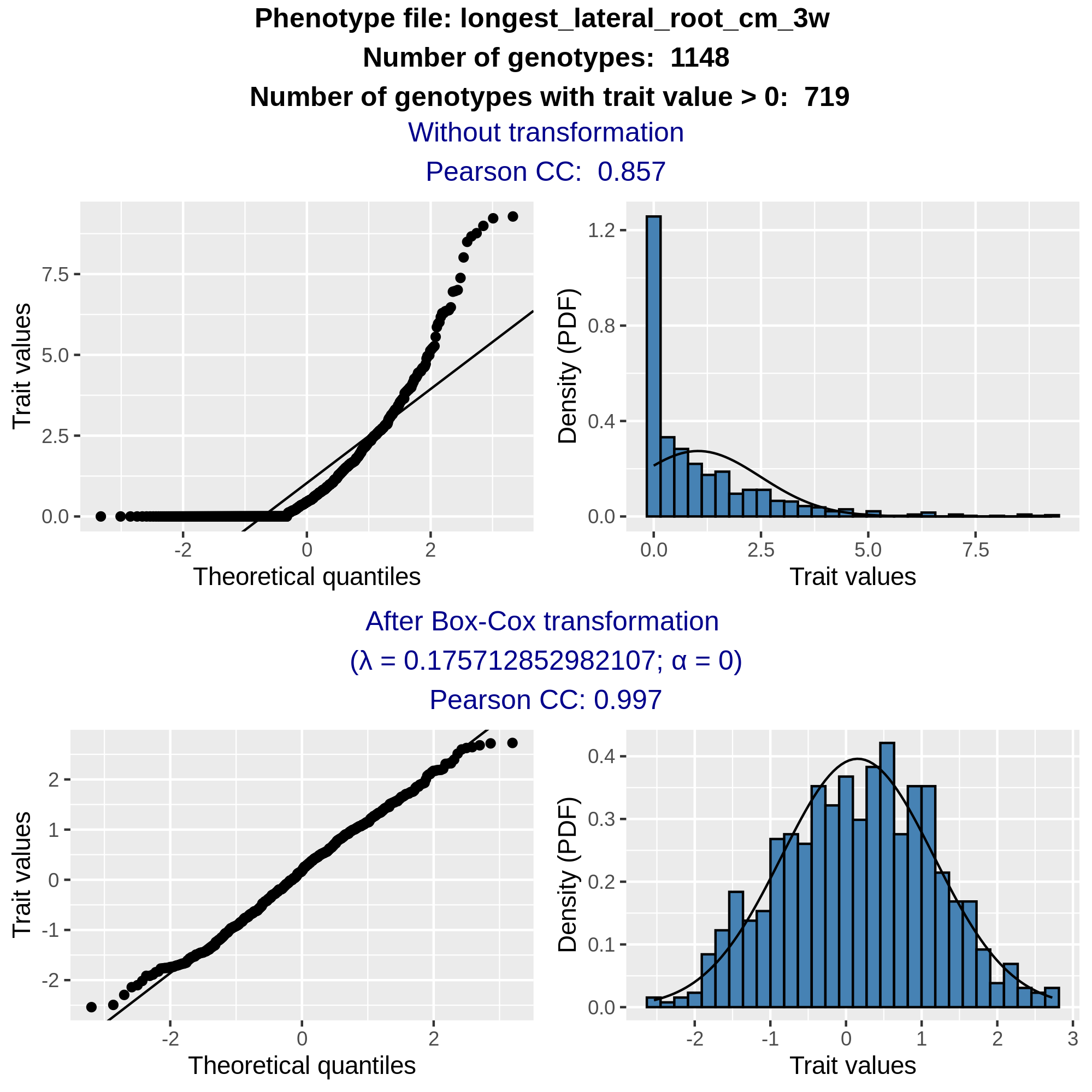

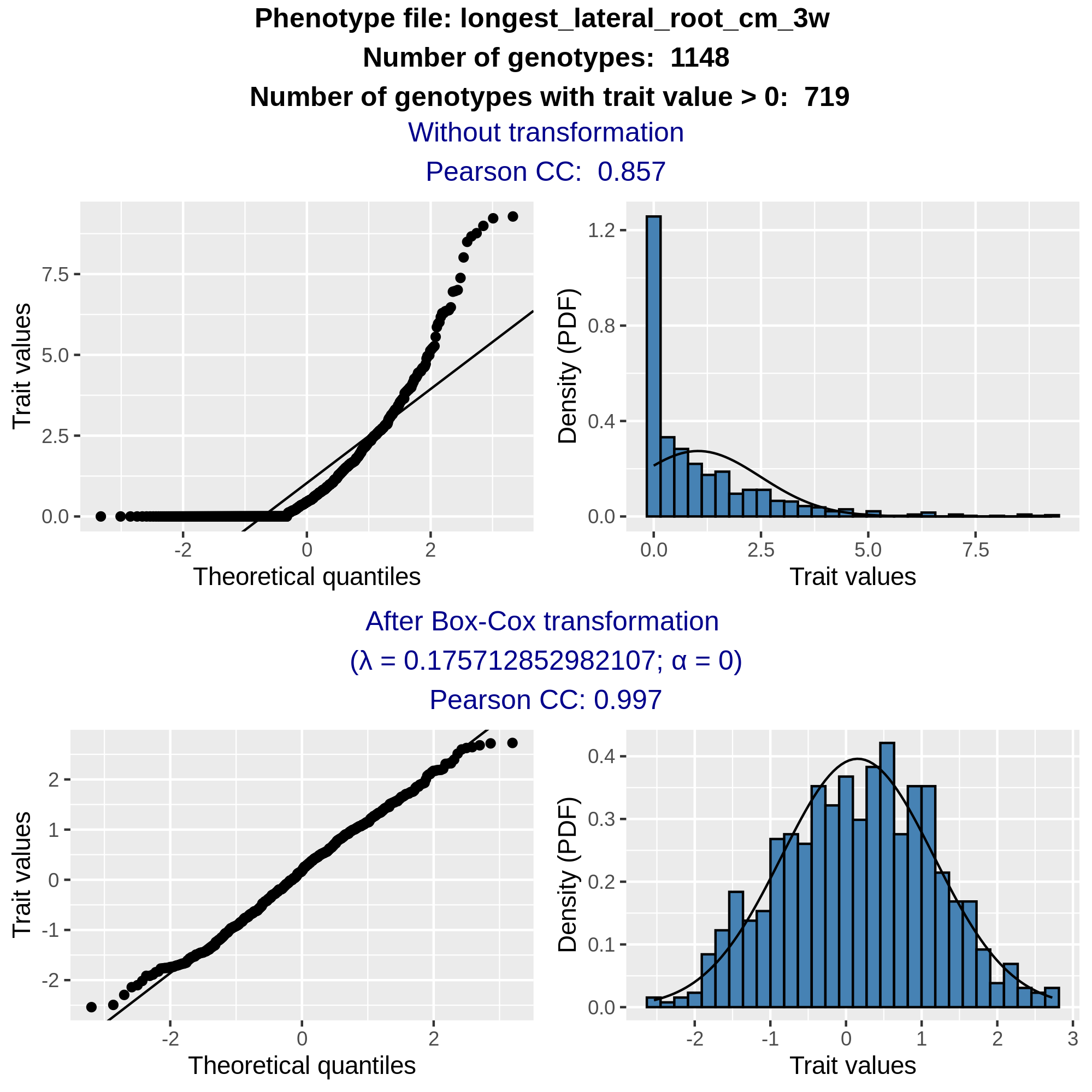

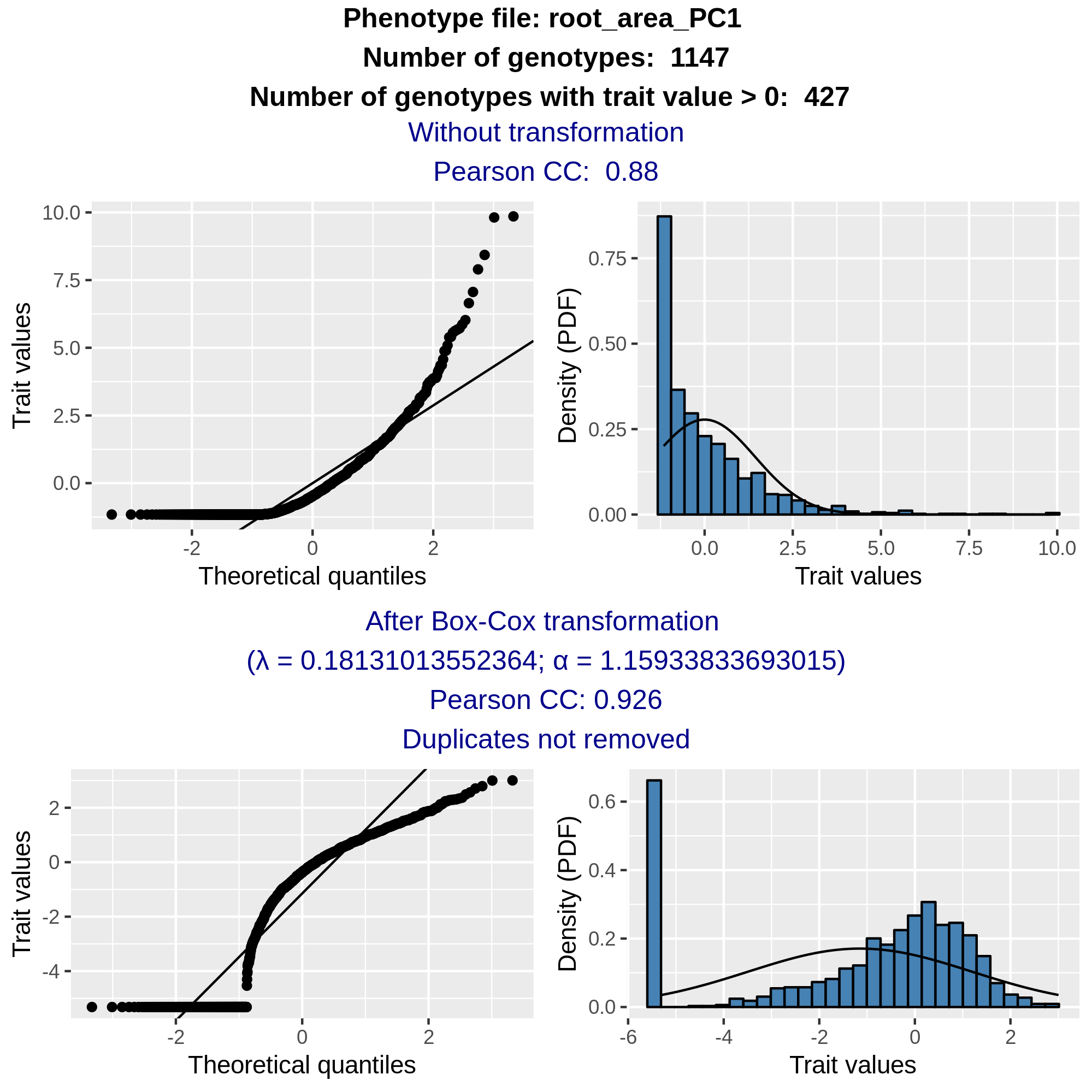

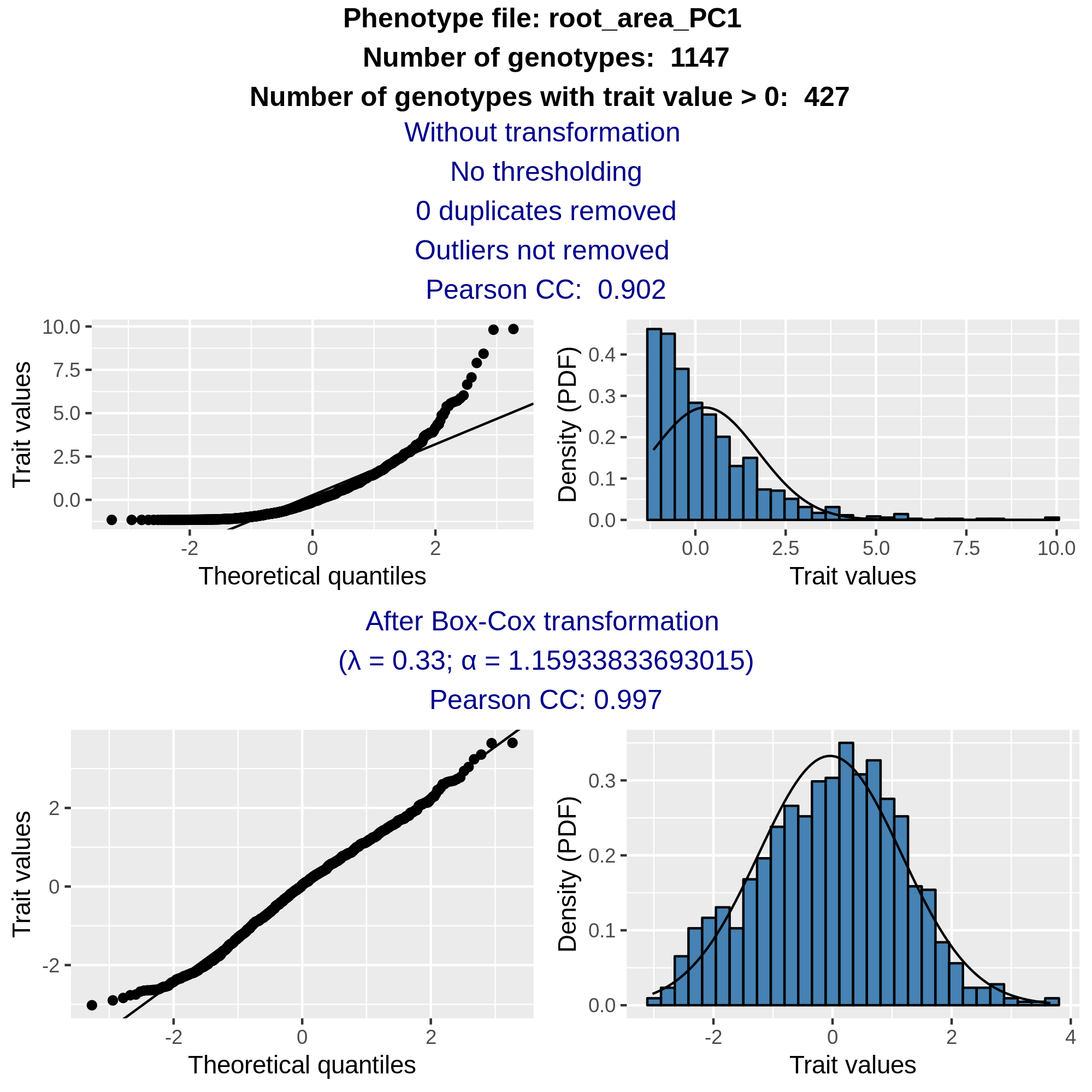


(A) (B)

(C) (D)

(E) (F)

(G) (H)

**Supplemental Figure 5.** Distributions of traits before and after transformations: **(A-D)** Longest lateral root at week 3 before transformation **(A, B)** and after transformation **(C, D)**. **(E-H)** Root area PC1 before transformation **(E, F)** and after transformation **(G, H)**. Q-Q plots represent the phenotype of a given genotype as a black dot and feature quantiles of a theoretical normal distribution with the same mean and variance as the observed data **(A, C, E, G)**. Histograms display the density of phenotypes via the probability density function (PDF) of the observed data, with the theoretical normal distribution superimposed as a black line **(B, D, F, H)**.
