# Supplementary material for: GWAS identifies candidate genes controlling adventitious rooting in Populus trichocarpa
Source: Hortic Res. 2023 Jun 14;10(8):uhad125. doi: 10.1093/hr/uhad125 (PMC10407606; doi:10.1093/hr/uhad125)
Supplement: Web_Material_uhad125 [file web_material_uhad125.zip › Note_on_S6-S9.docx]

Please note that Table S6-S9 were submitted through the system as “Supplementary File – Data Files” because these spreadsheets (.xslx format) are too large to be merged into the main PDF. Thank you.
