# Supplementary material for: GWAS identifies candidate genes controlling adventitious rooting in Populus trichocarpa
Source: Hortic Res. 2023 Jun 14;10(8):uhad125. doi: 10.1093/hr/uhad125 (PMC10407606; doi:10.1093/hr/uhad125)
Supplement: Web_Material_uhad125 [file web_material_uhad125.zip › Table_S1-S5.docx]

**Supplemental Table 1.** Timing of cutting collection and rooting assay are given for each experimental batch. This study was divided into eight batches due to limitations in our abilities to study all genotypes at once.

| GWAS Phase | Date of cutting collection from field | Start date of rooting assay |
| --- | --- | --- |
| 1 | 1/12-1/13/2018 | 3/21/18 |
| 2 | 1/12-1/13/2018 | 5/11/18 |
| 3 | 1/12-1/13/2018 | 7/27/18 |
| 4 | 1/12-1/13/2018 | 9/28/18 |
| 5 | 1/12-1/13/2018 | 11/13/18 |
| 6 | 1/12-1/13/2018 | 12/17/18 |
| 7 | 1/12-1/13/2018 | 2/4/19 |
| 8 | 2/6-2/7/2019 | 3/19/19 |

**Supplemental Table 2.** Raw traits obtained by parsing computer vision outputs into vectors with value of continuous trait for each genotype; all traits were transformed first by excluding zero values, and finally by a Box-Cox transformation. The Pearson correlation coefficient (CC) of each trait against a theoretical normal distribution is given before and after transformation. To aid visualization, conditional formatting in Excel was applied to color values from low (red) to high (green).

| *Trait category* | *Specific trait* | *Pearson CC of trait and normal dist.* | |
| --- | --- | --- | --- |
|  |  | *Before transf.* | *After transf.* |
| Basal root area | Growth from week 2-5 | 0.667 | 0.994 |
|  | Week 2 | 0.413 | 0.995 |
|  | Week 3 | 0.561 | 0.995 |
|  | Week 4 | 0.612 | 0.996 |
|  | Week 5 | 0.665 | 0.995 |
| Lateral root area | Growth from week 2-5 | 0.893 | 0.998 |
|  | Week 2 | 0.671 | 0.997 |
|  | Week 3 | 0.763 | 0.999 |
|  | Week 4 | 0.846 | 0.996 |
|  | Week 5 | 0.885 | 0.997 |
| Longest basal root | Week 2 | 0.502 | 0.994 |
|  | Week 3 | 0.653 | 0.995 |
|  | Week 4 | 0.696 | 0.992 |
|  | Week 5 | 0.746 | 0.996 |
| Longest lateral root | Week 2 | 0.766 | 0.997 |
|  | Week 3 | 0.857 | 0.998 |
|  | Week 4 | 0.905 | 0.995 |
|  | Week 5 | 0.933 | 0.997 |
| Longest root | Week 2 | 0.782 | 0.997 |
|  | Week 3 | 0.885 | 0.997 |
|  | Week 4 | 0.927 | 0.993 |
|  | Week 5 | 0.954 | 0.996 |
| Total root area | Growth from week 2-5 | 0.907 | 0.998 |
|  | Week 2 | 0.691 | 0.996 |
|  | Week 3 | 0.784 | 0.999 |
|  | Week 4 | 0.852 | 0.998 |
|  | Week 5 | 0.903 | 0.998 |

**Supplemental Table 3.** Principal component (PC) traits obtained by performing principal component analysis (PCA) to reduce large numbers of raw traits (Table S2) into a smaller number of variables. These traits were processed by a range of transformations, labeled in the “Transf. type” column. Transformation “A” simply involved excluding genotypes which have a zero value for all raw traits, followed by a Box-Cox transformation. Transformation “B” additionally featured removal of outliers prior to Box-Cox, while “C” also involved thresholding at an inflection point (elbow) prior to Box-Cox. The Pearson correlation coefficient (CC) of each trait against a theoretical normal distribution is given before and after transformation. To aid visualization, conditional formatting in Excel was applied to color values from low (red) to high (green).

| *Traits included in group for PCA* | *PC #* | *Transf. type* | *Pearson CC of trait and normal dist.* | |
| --- | --- | --- | --- | --- |
|  |  |  | Before transf. | After transf. |
| Eight root length traits: Longest root length across basal and lateral roots and all four timepoints | PC1 | A | 0.930 | 0.997 |
|  | PC2 | C | 0.790 | 0.993 |
|  | PC3 | B | 0.955 | 0.997 |
|  | PC4 | B | 0.869 | 0.981 |
| Eight root area traits: Root area across basal and lateral roots and all four timepoints | PC1 | A | 0.880 | 0.997 |
|  | PC2 | C | 0.687 | 0.984 |
|  | PC3 | B | 0.905 | 0.995 |
|  | PC4 | B | 0.775 | 0.980 |
| Sixteen root area and length traits: Root area and longest root length across basal and lateral roots and all four timepoints | PC1 | A | 0.879 | 0.999 |
|  | PC2 | B | 0.903 | 0.990 |
|  | PC3 | B | 0.775 | 0.974 |
|  | PC4 | B | 0.942 | 0.996 |

**Supplemental Table 4.** Narrow-sense SNP heritability (***h*^2^_SNP_**) is shown as computed by GEMMA. Standard error (SE) of this heritability estimate is also given. Treatments applied to principal component (PC) traits prior to transformation include thresholding, removal of duplicate values (corresponding to genotypes with zero values for all input traits), and removal of outliers, as described in Table S3. Transformations applied include the Box-Cox transformation and rank-based inverse (RB-INV) normal transformation.

| **Trait** | **Heritability** | | **Treatment of trait data before GWAS** | | | |
| --- | --- | --- | --- | --- | --- | --- |
|  | ***h*^2^_SNP_** | **SE(*h*^2^_SNP_)** | **Threshold** | **Removal of duplicate values** | **Removal of outliers** | **Transformation** |
| Root area PC2 | 0.218 | 0.084 | None | Y | N | RB-INV |
| Longest root length PC2 | 0.205 | 0.079 | None | Y | N | RB-INV |
| Total root area (wk. 5) | 0.141 | 0.094 | None | N | N | Box-Cox |
| Total root area growth (wk. 2 - wk. 5) | 0.138 | 0.089 | None | N | N | Box-Cox |
| Root area PC1 | 0.127 | 0.097 | None | Y | N | RB-INV |
| Root traits overall PC1 | 0.125 | 0.100 | None | Y | N | RB-INV |
| Root area PC1 | 0.123 | 0.096 | None | Y | N | Box-Cox |
| Longest root length PC1 | 0.120 | 0.094 | None | Y | N | RB-INV |
| Longest root length PC1 | 0.117 | 0.096 | None | Y | N | Box-Cox |
| Root traits overall PC1 | 0.117 | 0.098 | None | Y | N | Box-Cox |
| Basal root area growth (wk. 2 - wk. 5) | 0.114 | 0.135 | None | N | N | Box-Cox |
| Longest lateral root length (wk. 3) | 0.113 | 0.110 | None | N | N | Box-Cox |
| Basal root area (wk. 5) | 0.112 | 0.136 | None | N | N | Box-Cox |
| Root traits overall PC2 | 0.104 | 0.066 | None | Y | N | RB-INV |
| Total root area (wk. 3) | 0.100 | 0.146 | None | N | N | Box-Cox |
| Lateral root area (wk. 3) | 0.075 | 0.135 | None | N | N | Box-Cox |
| Root traits overall PC2 | 0.050 | 0.069 | None | Y | Y | Box-Cox |
| Longest lateral root length (wk. 2) | 0.044 | 0.135 | None | N | N | Box-Cox |
| Longest root length (wk. 5) | 0.037 | 0.120 | None | N | N | Box-Cox |
| Longest basal root length (wk. 5) | 0.021 | 0.108 | None | N | N | Box-Cox |
| Longest root length (wk. 2) | 0.020 | 0.228 | None | N | N | Box-Cox |
| Longest lateral root length (wk. 5) | 0.020 | 0.068 | None | N | N | Box-Cox |
| Basal root area (wk. 3) | 9.37 E-03 | 0.214 | None | N | N | Box-Cox |
| Longest root length PC2 | 5.36 E-03 | 0.078 | 0.708 | Y | Y | Box-Cox |
| Longest root length (wk. 3) | 1.66 E-03 | 0.128 | None | N | N | Box-Cox |
| Root area PC2 | 2.07 E-06 | 0.279 | 0.364 | Y | Y | Box-Cox |
| Lateral root area (wk. 5) | 2.04 E-06 | 0.062 | None | N | N | Box-Cox |
| Lateral root area growth (wk. 2- wk. 5) | 2.04 E-06 | 0.144 | None | N | N | Box-Cox |
| Longest lateral root length (wk. 4) | 2.04 E-06 | 0.057 | None | N | N | Box-Cox |
| Lateral root area (wk. 4) | 2.04 E-06 | 0.061 | None | N | N | Box-Cox |
| Lateral root area (wk. 2) | 2.04 E-06 | 0.092 | None | N | N | Box-Cox |
| Longest root length (wk. 4) | 2.03 E-06 | 0.078 | None | N | N | Box-Cox |
| Total root area (wk. 4) | 2.03 E-06 | 0.065 | None | N | N | Box-Cox |
| Total root area (wk. 2) | 2.03 E-06 | 0.075 | None | N | N | Box-Cox |
| Longest basal root length (wk. 3) | 1.96 E-06 | 0.235 | None | N | N | Box-Cox |
| Longest basal root length (wk. 4) | 1.96 E-06 | 0.220 | None | N | N | Box-Cox |
| Basal root area (wk. 4) | 1.96 E-06 | 0.411 | None | N | N | Box-Cox |
| Longest basal root length (wk. 2) | 1.95 E-06 | 0.331 | None | N | N | Box-Cox |
| Basal root area (wk. 2) | 1.95 E-06 | 2.447 | None | N | N | Box-Cox |

**Supplemental Table 5.** Tallies and statistics of QTL peaks found significant across methods, traits and significance thresholds. Traits studied include measures of root area, longest root length (LRL), and principal component (PC) traits computed over groups of traits. In cases where two MTMCSKAT associations in close proximity have the same *p*-value (1e-7) due to the limits of 10 million permutations, the position nearest to the respective gene was used for these calculations.

| **Grouping of QTLs by significance and distance** | **Trait** | **Method** | **N genes** | **Positions of QTL peaks relative to nearest transcript** | | | | |
| --- | --- | --- | --- | --- | --- | --- | --- | --- |
|  |  |  |  | **Avg. distance (bp)** | **Median distance (bp)** | **Percent intergenic** | **Percent upstream** | **Percent downstream** |
| All QTLs passing Bonf. | Basal area growth (wk. 2-5) | MTMCSKAT | 6 | 1,648 | 195 | 50% | 33% | 17% |
|  | Longest lateral root  (wk. 3) | MTMCSKAT | 13 | 8,464 | 3,423 | 77% | 23% | 54% |
|  | LRL PC2 | MTMCSKAT | 1 | 0 | 0 | 0% | 0% | 0% |
|  | Root area PC1 | MTMCSKAT | 9 | 1,722 | 323 | 67% | 44% | 22% |
|  | Root area PC2 | MTMCSKAT | 4 | 691 | 365 | 50% | 50% | 0% |
|  | Root traits overall PC1 | MTMCSKAT | 1 | 35,938 | 35,938 | 100% | 100% | 0% |
|  | Root traits overall PC2 | MTMCSKAT | 1 | 21,355 | 21,355 | 100% | 0% | 100% |
|  | Basal root area (wk. 5) | MTMCSKAT | 6 | 1,770 | 560 | 67% | 50% | 17% |
|  | Total root area (wk. 5) | MTMCSKAT | 1 | 9,499 | 9,499 | 100% | 0% | 100% |
| All QTLs passing Bonf. within 5kb of gene | Basal area growth (wk. 2-5) | MTMCSKAT | 5 | 485 | 0 | 40% | 20% | 20% |
|  | Longest lateral root  (wk. 3) | MTMCSKAT | 9 | 1,805 | 1,181 | 67% | 33% | 33% |
|  | LRL PC2 | MTMCSKAT | 1 | 0 | 0 | 0% | 0% | 0% |
|  | Root area PC1 | MTMCSKAT | 8 | 628 | 188 | 63% | 38% | 25% |
|  | Root area PC2 | MTMCSKAT | 4 | 691 | 365 | 50% | 50% | 0% |
|  | Basal root area (wk. 5) | MTMCSKAT | 5 | 631 | 390 | 60% | 40% | 20% |
| All QTLs passing FDR (alpha = 0.10) and/or Bonf. | Basal area growth (wk. 2-5) | MTMCSKAT | 28 | 13,475 | 3,206 | 68% | 43% | 25% |
|  | Longest lateral root  (wk. 3) | GEMMA | 2 | 8,837 | 8,837 | 100% | 50% | 50% |
|  | Longest lateral root  (wk. 3) | MTMCSKAT | 65 | 6,078 | 1,936 | 77% | 40% | 37% |
|  | LRL PC1 | MTMCSKAT | 10 | 3,254 | 2,996 | 80% | 40% | 40% |
|  | LRL PC2 | MTMCSKAT | 5 | 16,432 | 5,659 | 80% | 40% | 40% |
|  | Root area PC1 | MTMCSKAT | 29 | 5,837 | 1,437 | 69% | 34% | 34% |
|  | Root area PC2 | MTMCSKAT | 26 | 10,690 | 2,470 | 77% | 46% | 31% |
|  | Root traits overall PC1 | MTMCSKAT | 29 | 12,084 | 1,942 | 66% | 34% | 31% |
|  | Root traits overall PC2 | MTMCSKAT | 6 | 4,129 | 617 | 83% | 0% | 83% |
|  | Basal root area (wk. 5) | MTMCSKAT | 21 | 10,773 | 6,570 | 86% | 57% | 29% |
|  | Total root area (wk. 5) | MTMCSKAT | 2 | 5,938 | 5,938 | 100% | 50% | 50% |
| All QTLs passing FDR (alpha = 0.10) and/or Bonf. within 5kb of gene | Basal area growth (wk. 2-5) | MTMCSKAT | 17 | 1,050 | 0 | 47% | 35% | 12% |
|  | Longest lateral root  (wk. 3) | MTMCSKAT | 48 | 1,350 | 617 | 69% | 40% | 29% |
|  | LRL PC1 | MTMCSKAT | 7 | 1,565 | 608 | 71% | 43% | 29% |
|  | LRL PC2 | MTMCSKAT | 2 | 2,484 | 2,484 | 50% | 50% | 0% |
|  | Root area PC1 | MTMCSKAT | 23 | 1,267 | 575 | 61% | 39% | 22% |
|  | Root area PC2 | MTMCSKAT | 16 | 1,076 | 411 | 63% | 38% | 25% |
|  | Root traits overall PC1 | MTMCSKAT | 18 | 900 | 0 | 44% | 28% | 17% |
|  | Root traits overall PC2 | MTMCSKAT | 5 | 684 | 396 | 80% | 0% | 80% |
|  | Basal root area (wk. 5) | MTMCSKAT | 10 | 938 | 560 | 70% | 50% | 20% |
|  | Total root area (wk. 5) | MTMCSKAT | 1 | 2,377 | 2,377 | 100% | 100% | 0% |
| All QTLs passing ART-Bonf. | Basal area growth (wk. 2-5) | GMMAT | 4 | 4,127 | 3,118 | 100% | 50% | 50% |
|  | Total area growth (wk. 2-5) | GMMAT | 3 | 1,444 | 840 | 100% | 67% | 33% |
|  | Root area PC2 | GMMAT | 1 | 1,695 | 1,695 | 100% | 0% | 100% |
|  | Basal area growth (wk. 2-5) | GEMMA | 11 | 3,583 | 1,696 | 91% | 36% | 55% |
|  | Total root area growth (wk. 2-5) | GEMMA | 17 | 2,445 | 946 | 82% | 29% | 53% |
|  | Longest lateral root  (wk. 3) | GEMMA | 11 | 3,163 | 1,932 | 91% | 27% | 64% |
|  | LRL PC1 | GEMMA | 13 | 2,800 | 2,740 | 100% | 54% | 46% |
|  | LRL PC2 | GEMMA | 15 | 3,490 | 2,893 | 87% | 53% | 33% |
|  | Root area PC1 | GEMMA | 16 | 2,335 | 1,712 | 88% | 44% | 44% |
|  | Root area PC2 | GEMMA | 17 | 2,104 | 528 | 82% | 29% | 53% |
|  | Root traits overall PC1 | GEMMA | 4 | 3,105 | 3,068 | 100% | 75% | 25% |
|  | Root traits overall PC2 | GEMMA | 11 | 4,665 | 2,130 | 100% | 36% | 64% |
|  | Basal root area (wk. 5) | GEMMA | 12 | 3,658 | 2,210 | 83% | 42% | 42% |
|  | Total root area (wk. 5) | GEMMA | 7 | 1,791 | 777 | 86% | 43% | 43% |
| All QTLs passing ART-Bonf. within 5kb of gene | Basal area growth (wk. 2-5) | GMMAT | 2 | 987 | 987 | 100% | 50% | 50% |
|  | Total area growth (wk. 2-5) | GMMAT | 3 | 1,444 | 840 | 100% | 67% | 33% |
|  | Root area PC2 | GMMAT | 1 | 1,695 | 1,695 | 100% | 0% | 100% |
|  | Basal area growth (wk. 2-5) | GEMMA | 7 | 1,235 | 1,109 | 86% | 29% | 57% |
|  | Total root area growth (wk. 2-5) | GEMMA | 14 | 1,258 | 898 | 79% | 29% | 50% |
|  | Longest lateral root  (wk. 3) | GEMMA | 8 | 1,575 | 1,578 | 88% | 38% | 50% |
|  | LRL PC1 | GEMMA | 12 | 2,117 | 2,293 | 100% | 58% | 42% |
|  | LRL PC2 | GEMMA | 11 | 2,051 | 1,892 | 82% | 55% | 27% |
|  | Root area PC1 | GEMMA | 15 | 1,823 | 1,579 | 87% | 40% | 47% |
|  | Root area PC2 | GEMMA | 15 | 1,081 | 505 | 80% | 33% | 47% |
|  | Root traits overall PC1 | GEMMA | 3 | 2,085 | 2,737 | 100% | 67% | 33% |
|  | Root traits overall PC2 | GEMMA | 8 | 1,684 | 1,536 | 100% | 25% | 75% |
|  | Basal root area (wk. 5) | GEMMA | 7 | 1,004 | 1,041 | 71% | 29% | 43% |
|  | Total root area (wk. 5) | GEMMA | 6 | 1,101 | 586 | 83% | 33% | 50% |
